# Dopant redox stability as a design principle for selective CO_2_ electroreduction on Cu

**DOI:** 10.1039/d6cp01600f

**Published:** 2026-07-30

**Authors:** Francesco Barberini, Antonio Barile, Paolo Restuccia, Pierfrancesco Atanasio, Daniele Passeri, Devis Di Tommaso, Raffaello Mazzaro, Luca Pasquini, M. Clelia Righi

**Affiliations:** a Department of Physics and Astronomy, University of Bologna Viale Berti Pichat 6/2 40127 Bologna Italy paolo.restuccia@unibo.it raffaello.mazzaro@unibo.it; b Department of Basic and Applied Sciences for Engineering, Sapienza University of Rome Via A. Scarpa 14 00161 Roma Italy; c School of Physical and Chemical Sciences, Queen Mary University of London Mile End Road 327 E1 4NS London UK

## Abstract

The rational design of selective Cu-based electrocatalysts for CO_2_ electroreduction requires an understanding of how dopants modify catalytic pathways under realistic conditions. However, the active surface often evolves dynamically during electrolysis, hindering direct comparison between theoretical predictions and experimental selectivity. Herein, we investigate how dopant redox stability influences reaction pathways on Cu by combining controlled sputter-based synthesis of Ti- and Sn-modified Cu thin films with electrochemical measurements and dispersion-corrected density functional theory (DFT-D) within the computational hydrogen electrode framework. Composition-spread electrodes enable systematic comparison of dopant effects, while DFT-D quantifies how Ti and Sn modify the thermodynamics of key intermediates (H*, COOH*, CO*, and OCHO*). Experiments reveal that Ti-modified Cu favors hydrogen evolution, whereas Sn-modified Cu produces CO with unaccounted charge consistent with liquid products. Post-electrolysis XRD shows TiO_2_ formation on Cu@Ti and Cu_3_Sn intermetallic phases on Cu@Sn, indicating distinct phase evolution during reaction. Combined experimental-theoretical analysis reveals a stability–selectivity relationship in doped Cu catalysts: oxophilic Ti oxidizes into HER-active oxide motifs, whereas redox-stable Sn forms metallic or intermetallic environments that stabilize O-bound intermediates and promote CO/formate pathways. This experimentally validated framework provides a transferable guideline for designing Cu-based CO_2_ reduction catalysts and may enable future high-throughput screening of dopant chemistries.

## Abstract 2

Different dopants can govern the selectivity of Cu-based CO_2_RR catalysts: Ti promotes oxidation and H-bound states, favoring H_2_ evolution, while Sn promotes alloying and O-bound intermediates, steering selectivity toward CO and formic acid.

## Graphical abstract

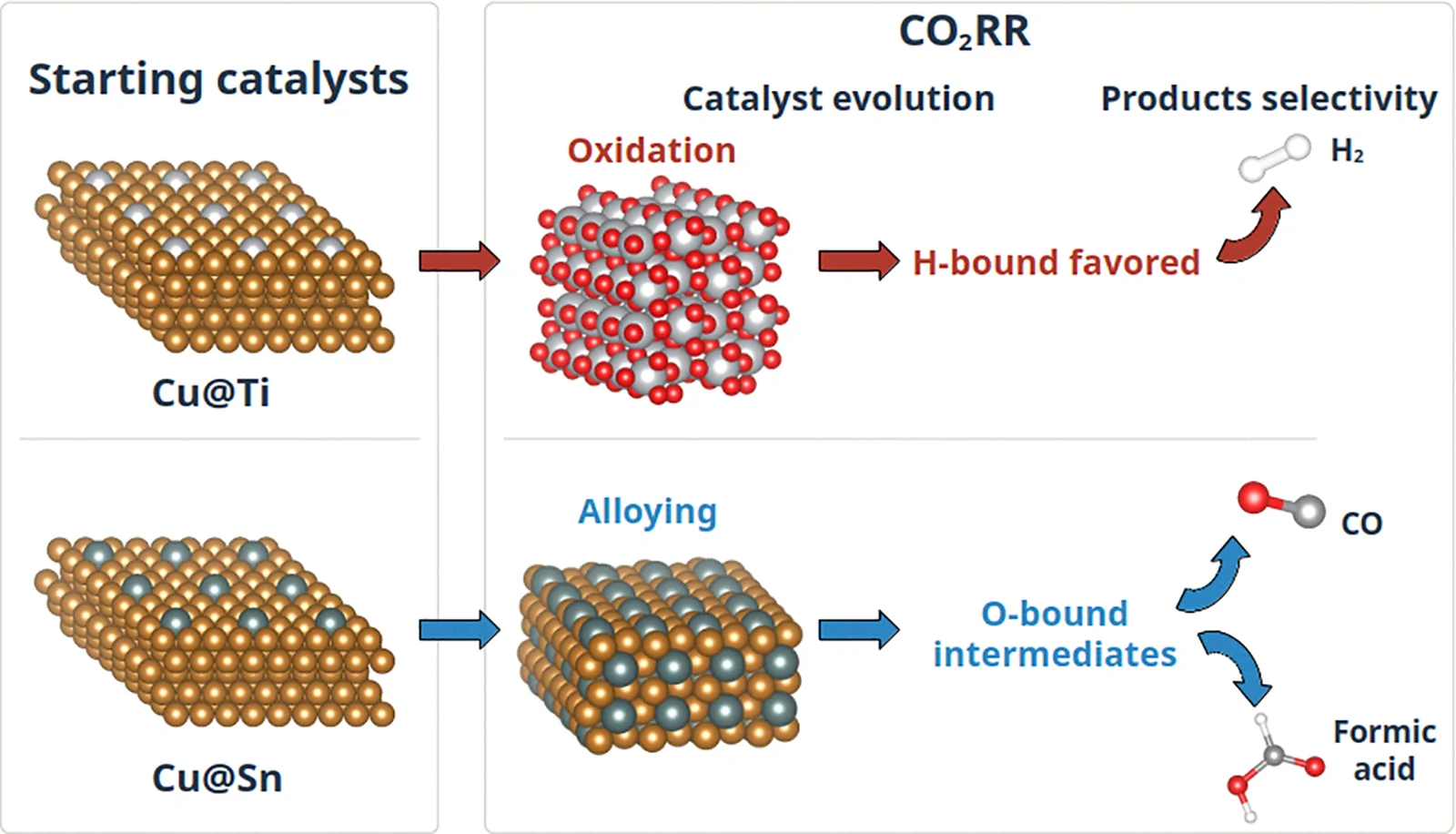

## 1 Introduction

The rapid increase in atmospheric CO_2_ and the consequent global warming have led to air pollution, ocean acidification,^1^ and climate change, prompting the scientific community to develop strategies for carbon capture and utilization. In particular, the conversion of CO_2_ into valuable products through reduction processes offers a sustainable and potentially economically viable solution.^2–4^ One of the most promising technologies in this field is the electrochemical reduction of carbon dioxide, or the CO_2_ reduction reaction (CO_2_RR), which enables the conversion of CO_2_ into fuels and chemicals.

To achieve this target, the choice of an appropriate electrocatalytic substrate is paramount. Among the many metal-based electrocatalysts, copper stands out as one of the most promising materials due to its ability to reduce CO_2_ to various products, such as hydrocarbons, aldehydes, and alcohols,^5–7^ yet this same versatility makes product selectivity difficult to control. In practice, small variations in local composition, surface structure, electrolyte environment, and applied potential can strongly alter the competition between CO_2_ reduction and the hydrogen evolution reaction (HER). As a result, the rational design of Cu-based catalysts still depends on establishing a clearer connection between atomistic surface properties and experimentally observed selectivity. Indeed, while macro-scale experiments allowed discovering the efficiency and selectivity of these catalytic structures,^3,8^ a comprehensive microscopic description of the chemical reactions occurring at the electrocatalytic surfaces is still lacking due to the multitude of concurrent factors influencing the catalytic process. In this context, *ab initio* simulations, such as those based on density functional theory (DFT), serve as powerful tools for linking atomistic properties to macroscopic quantities in CO_2_RR experiments, including current densities, overpotentials, and faradaic efficiency.^9,10^ Integrating computational modelling with experimental studies is promising for designing catalytic substrates with tailored properties and accelerating the development of efficient CO_2_ conversion and utilization strategies.^11^

One widely explored strategy to tune Cu reactivity is the introduction of a second metal through doping or alloying. However, predictive design remains challenging because the catalytically active surface under operating conditions often differs from the nominal as-prepared material. Dopants can segregate, oxidize, alloy, or reconstruct during electrolysis, thereby changing both the nature of the active sites and the energetics of key reaction intermediates. Under these conditions, selectivity cannot be understood solely from equilibrium adsorption trends on idealized structures. A reliable comparison between experiment and theory, therefore, requires model systems and measurements that account for the dynamic chemical state of the catalyst under bias.

In this context, Ti and Sn provide a particularly informative contrast. Ti is strongly oxophilic and is expected to undergo oxidation and surface reconstruction under CO_2_ reduction conditions. Nevertheless, the combination of these two metals can give rise to unexpected catalytic behavior. For example, Lu *et al.* demonstrated that a hierarchical nanoporous Cu–Ti bimetallic electrocatalyst achieved hydrogen evolution rates more than twice those of commercial carbon-supported Pt under mild overpotentials.^12^ The synergistic interaction between Cu and Ti has also been exploited to improve CO_2_ electroreduction. For instance, Stalinraja *et al.* reported Cu-electrodeposited TiO_2_ nanotubes that exhibited a faradaic efficiency of approximately 55% toward C_2_H_4_, significantly outperforming bare TiO_2_ nanotubes. Combined experimental and DFT analyses revealed that Cu deposition on the TiO_2_ surface modifies the adsorption energies of key reaction intermediates, promoting C–C coupling and favoring ethylene formation over competing pathways.^13^ More recently, Zhang *et al.* have theoretically investigated Ti@Cu single-atom alloys for the electrochemical CO_2_ reduction reaction and demonstrated that isolated Ti atoms embedded within a Cu matrix can substantially alter the reaction energetics. Their study further showed that the oxidation state of the Ti active site, modulated by adsorbed oxygen species, plays a critical role in determining both catalytic activity and product selectivity, emphasizing the importance of the local Cu–Ti coordination environment under reaction conditions.^14^

As for Sn, it is more likely to remain in metallic or intermetallic Cu–Sn environments that can alter the balance between hydrogen adsorption and CO_2_ reduction intermediates.^15^ A study by Vasileff *et al.* showed that selectivity shifts from CO to formate generation as Sn content in the alloy catalyst increases due to a change in adsorption preference from the C-bound *COOH intermediate to the O-bound *OCHO intermediate. Theoretical DFT calculation results indicate that this selectivity shift is due to a gradual weakening of *COOH adsorption and strengthening of *OCHO that occurs with increasing Sn content.^16^ Another study by Sarfraz *et al.* demonstrated that decorating oxide-derived Cu with an optimal amount of Sn resulted in the formation of a Cu–Sn bimetallic catalyst that achieved CO faradaic efficiencies exceeding 90% over a wide potential range. The enhanced performance was attributed to the electronic interaction between Cu and Sn, which optimized the adsorption energies of key CO_2_RR intermediates while suppressing the competing hydrogen evolution reaction.^17^

Comparing these two dopants allows us to test the broader hypothesis that CO_2_RR selectivity on doped Cu is governed not only by the local adsorption properties of the nominal alloy but also by the thermodynamic stability of the dopant under operating conditions. In this view, alloy/phase stability and electrochemical Pourbaix stability must be considered together with adsorption-energy descriptors because they determine whether the dopant remains accessible in a metallic or intermetallic Cu–M environment or evolves toward oxide-rich motifs that modify the balance between the CO_2_RR and the HER. The proposed stability–selectivity relationship should therefore be regarded as a design principle to be tested across the broader Cu–M compositional space, where the outcome will depend on alloy thermodynamics, dopant segregation, oxide reducibility, Pourbaix stability, and the local coordination of the active surface under bias.

Here, we combine controlled sputter-based preparation of Cu, Ti-modified Cu, and Sn-modified Cu thin films with electrochemical measurements and dispersion-corrected DFT calculations within the computational hydrogen electrode (CHE) framework to establish a direct comparison between experimental behaviour and theoretical reaction energetics. We use DFT–CHE calculations to resolve why Ti- and Sn-doped Cu diverge in the HER *versus* CO_2_RR selectivity by quantifying how each dopant reshapes the thermodynamics of H*, COOH*, CO*, and OCHO* across Cu(111), Ti(0001), Sn(111), and Cu single atom alloy (SAA) models (Cu@Ti, Cu@Sn).

This approach allows us to identify how Ti and Sn reshape the thermodynamics of the key intermediates H*, COOH*, CO*, and OCHO*, and to relate these trends to the phases that emerge under reaction conditions. We show that Ti drives the catalyst toward the HER through oxidation and reconstruction into Ti-containing oxide motifs, whereas Sn favours metallic or intermetallic Cu–Sn environments that stabilize O-bound intermediates and redirect selectivity toward CO/formate pathways. On this basis, we propose a stability–selectivity relationship for Cu-based CO_2_ reduction catalysts, in which dopant redox stability determines whether the surface remains accessible to CO_2_ reduction pathways or evolves toward HER-active states. More broadly, this experimentally validated framework could provide a robust basis for future high-throughput screening of Cu-based catalyst libraries.

## 2 Methods

### 2.1 Experimental methods

The Cu, Cu@Ti, and Cu@Sn electrodes were fabricated using magnetron co-sputtering (MS). This approach enabled the simultaneous deposition of two distinct materials (*e.g.*, copper and titanium or copper and tin) along separate directions, resulting in electrodes characterized by both thickness and concentration gradients. This method enables continuous and precise control of catalyst composition on a single substrate, allowing systematic evaluation of dopant effects without changes in synthesis conditions. Compared to conventional alloy or wet-chemical methods, it minimizes variability in morphology and phase segregation while providing clean, well-defined interfaces and access to metastable compositions relevant for CO_2_RR optimization. To obtain the best configuration in Cu@Ti and Cu@Sn heterostructures, a wide range of compositions was achieved due to the steradian distribution of the sputtered flux under stationary substrate conditions. Once the best composition was selected, MS was performed in a rotating sample holder configuration to achieve a homogeneous layer under a 10 standard cubic centimeters per minute (sccm) argon flow rate and a chamber pressure of 5 × 10^−3^ mbar. The deposition time was 20 min for the pure Cu electrode and 120 min for the alloyed Cu@Ti and Cu@Sn samples. The DC power applied to the Cu target was 80 W, while the RF powers used for the Cu, Ti, and Sn targets were 80 W, 20 W, and 15 W, respectively. To enhance mechanical adhesion and address challenges associated with the delamination of metallic copper under electrochemical testing conditions, oxygen plasma treatment was carried out on the substrate to clean the surface and activate it for better adhesion, and, subsequently, a 42 nm gold–chromium (Au–Cr) interlayer was deposited *via* evaporation between the fluorine-doped tin oxide (FTO) substrate and the sputtered material, consisting of a 7 nm chromium layer and a 35 nm gold layer.

Morphological and compositional analysis of the electrodes was performed by cross-sectional imaging and energy-dispersive X-ray spectroscopy (EDS) *via* scanning electron microscopy (SEM) Stereo Scan 360. The targeted atomic concentration ranges were chosen to be 2–15 at% Ti for Cu@Ti electrodes and 2–20 at% Sn for Cu@Sn electrodes,^12,16^ while the thickness was estimated to be 800 nm and 680 nm, respectively.

Electrochemical characterization of the gradient samples was performed with a customized scanning photoelectrochemical flow cell (SPFC),^18^ allowing for high-throughput screening of the entire electrode in a single experimental run, and identifying the best-performing alloy composition. The cell has an operating surface area of 1.5 mm in diameter and allows for a three-electrode configuration, using a platinum wire as the counter electrode (CE) and an Ag/AgCl electrode as the reference electrode (RE). The cell was operated with a continuous flow of CO_2_-saturated 0.5 M sodium bicarbonate (NaHCO_3_) as aqueous electrolyte, with pH adjusted to 8.5.

Using an AutoLab PGSTAT204 potentiostat, each analysis consisted of an open circuit potential (OCP) measurement, electrochemical impedance spectroscopy (EIS), and a two-cycle cyclic voltammetry (CV) scan. The CV measurements were conducted within a potential range of [−2, −1.6] V for the Cu@Ti electrode and [−1.6, −0.5] V for the Cu@Sn electrode. These values can be converted to RHE potentials *E*_RHE_, according to

1 *E*_RHE_ = *E*_Ag/AgCl_ + 0.197 V + 0.059 V·pH − *iR*

where *E*_Ag/AgCl_ is the working potential, and *iR* is the ohmic compensation term.

To determine the optimal electrode configuration, the overpotential at −10 mA cm^−2^ was used as a performance metric, as this parameter reflects the additional energy required to drive a reaction beyond its equilibrium potential, often due to kinetic barriers or side reactions. The lowest overpotential values, indicative of improved electrochemical activity, were obtained for Cu_95_Ti_05_ and Cu_90_Sn_10_ electrodes.

X-ray diffraction (XRD) measurements were performed prior to electrochemical testing using a Bruker D8 ADVANCE diffractometer equipped with a molybdenum (Mo) X-ray tube (*λ* = 0.71073 Å). Data were collected in Bragg–Brentano geometry in the 2*θ* range 10°–40°, with a step size of 0.0204°, operating at 40 kV and 30 mA. Phase identification was carried out using the Match! software. The analysis covered a pure copper sample as a reference, a titanium concentration gradient to observe the role of Ti in the electrode (including also the Cu_95_Ti_05_ configuration), and finally the Cu_90_Sn_10_ sample. The relative strain (*ε*) was calculated as:2
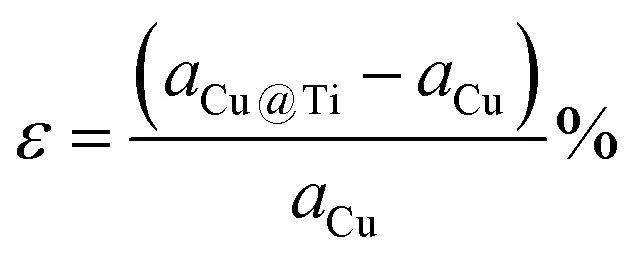
where *a*_Cu@Ti_ is the experimentally determined lattice parameter for each copper/titanium alloy, and *a*_Cu_ is the lattice parameter of pure copper. A second XRD profile was obtained using an X'Pert Pro Panalytical Bragg–Brentano diffractometer equipped with an X'Celerator multichannel solid-state detector and a source of Cu Kα radiation (*λ* = 1.5418 Å). Data were collected over the 2*θ* range 10°–70°.

CO_2_ conversion experiments were performed on a two-compartment cell. Here, the 0.5 M NaHCO_3_ solution was initially purged with a 1 sccm flow of CO_2_ and then pumped at 10 mL min^−1^ into a customised two-compartment flow cell, ensuring that the catholyte, along with the products of the reaction, was continuously recirculated back into a sealed reservoir, whose headspace is used to accumulate gas products. A Nafion ion exchange membrane was applied to separate the two compartments. Chronopotentiometric measurements were conducted in a three-electrode configuration, using a Gamry Interface 1010 potentiostat, an Hg/Hg_2_SO_4_ electrode and a platinum wire as the reference and counter electrodes, respectively. These values were also converted to RHE potentials *E*_RHE_, according to

3 *E*_RHE_ = *E*_Hg/Hg_2_SO_4__ + 0.640  V + 0.059 V·pH − *iR*

where *E*_Hg/Hg_2_SO_4__ is the working potential.

Different current densities (1, 5, and 10 mA cm^−2^) were analysed, while the produced gases were sampled at 10-minute intervals until the peak areas stabilized over time.

An Agilent 990 microgas chromatograph (μ-GC) was employed to characterize the gaseous products. Gaseous samples were analysed using two distinct columns: a Molsieve 5 Å and a Poraplot Q column. The μ-GC is equipped with a thermal conductivity detector (TCD) and operated with He as the gas carrier. A mixture of CO_2_ and syngas (with 50 : 50 volumetric proportion of H_2_/CO) was used to calibrate the instrument and establish a correlation between the measured peak area and the actual mole fraction of the gases in the mixture. To obtain multiple calibration points, the CO_2_ flow was kept constant at 45 sccm using an analogical flowmeter, while the syngas was varied to 1, 5, 10, 20, 25, and 40 sccm. The digital MFC flowmeter of MKS Instruments (range 50 sccm) was pre-calibrated with nitrogen; however, as both H_2_ and CO have a conversion factor of 1, no further adjustments were required. The calibration curves were fitted based on the peak areas recorded by the Molsieve column, while the Poraplot Q column was used to quantify CO_2_ in the mixture, as the Molsieve column does not detect carbon dioxide. The operating parameters for the two chromatographic columns were optimized to ensure distinct peak resolution. The injector temperature was set to 50 °C, the column temperature to 40 °C, and the carrier gas pressure to 150 kPa. Injection times were configured at 40 ms for the Molsieve column and 100 ms for the Poraplot Q column to enhance detection efficiency and peak separation.

A platinum electrode was employed to calibrate the setup for 100% faradaic efficiency towards hydrogen production. The FE of hydrogen for the other electrodes was calculated by dividing the mole percentage of H_2_ obtained from the calibration curve by that of the platinum reference. Additionally, a silver electrode was employed due to the known selectivity towards H_2_ and CO to further assess the reliability of the setup. This approach enabled the calculation of the faradaic efficiencies for all tested materials.

Finally, a comparison with the nominally produced gases was conducted by determining the ratio between the produced gas volume and the carrier gas flow, both in sccm. The volume of gas produced per minute (m^3^ min^−1^) was calculated using the ideal gas law:

4 *PV* = *nRT*

where *T* is room temperature, *P* is atmospheric pressure, *R* is the ideal gas constant (8.314 J K^−1^ mol^−1^), and *n* is the molar gas flow rate (mol min^−1^). The latter is obtained using Faraday's law:5
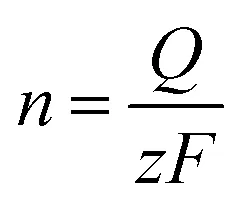
where *F* is the Faraday constant equal to 96 485 C mol^−1^, *z* is the number of electrons transferred per mole of produced gas, and *Q* is the total charge passed.

### 2.2 Computational methods

The DFT calculations were performed using the pseudopotential plane wave programs included in the Quantum Espresso (QE) package^19–21^ and in the Vienna *ab initio* Simulation Package (VASP),^22–24^ using ultrasoft^25^ and projector-augmented-wave^26^ pseudopotentials for the QE and VASP calculations, respectively. In particular, we employed QE for the structural optimisation and VASP to compute the zero-point energy and entropic contributions of the Gibbs free energy. This workflow was adopted to ensure compatibility with the available computational tools. In particular, Xsorb, used to identify the most stable molecular adsorption configurations, is interfaced with QE, whereas VASP was employed to evaluate vibrational contributions in our systems due to its more robust framework for phonon calculations. The exchange correlation functional was described by the GGA-PBE approximation.^27^ The long-range interactions were included in the form of van der Waals corrections within the Grimme-D3 scheme.^28^ The calculations were performed in a spin-polarized setup. A Gaussian smearing of 0.26 eV and 0.2 eV was also employed in the QE and VASP calculations, respectively.

For the bare substrates, we performed the calculations using a 2 × 2 in-plane supercell. We sampled the Brillouin zone of the Cu(111) supercell with a 9 × 9 × 1 **k**-point mesh grid^29^ and an energy cut-off of 40 Ry. For Ti(0001) and Sn(111), a 10 × 10 × 1 and a 6 × 6 × 1 **k**-point mesh grids were used, respectively, with an energy cut-off of 50 Ry.

**Fig. 1:**
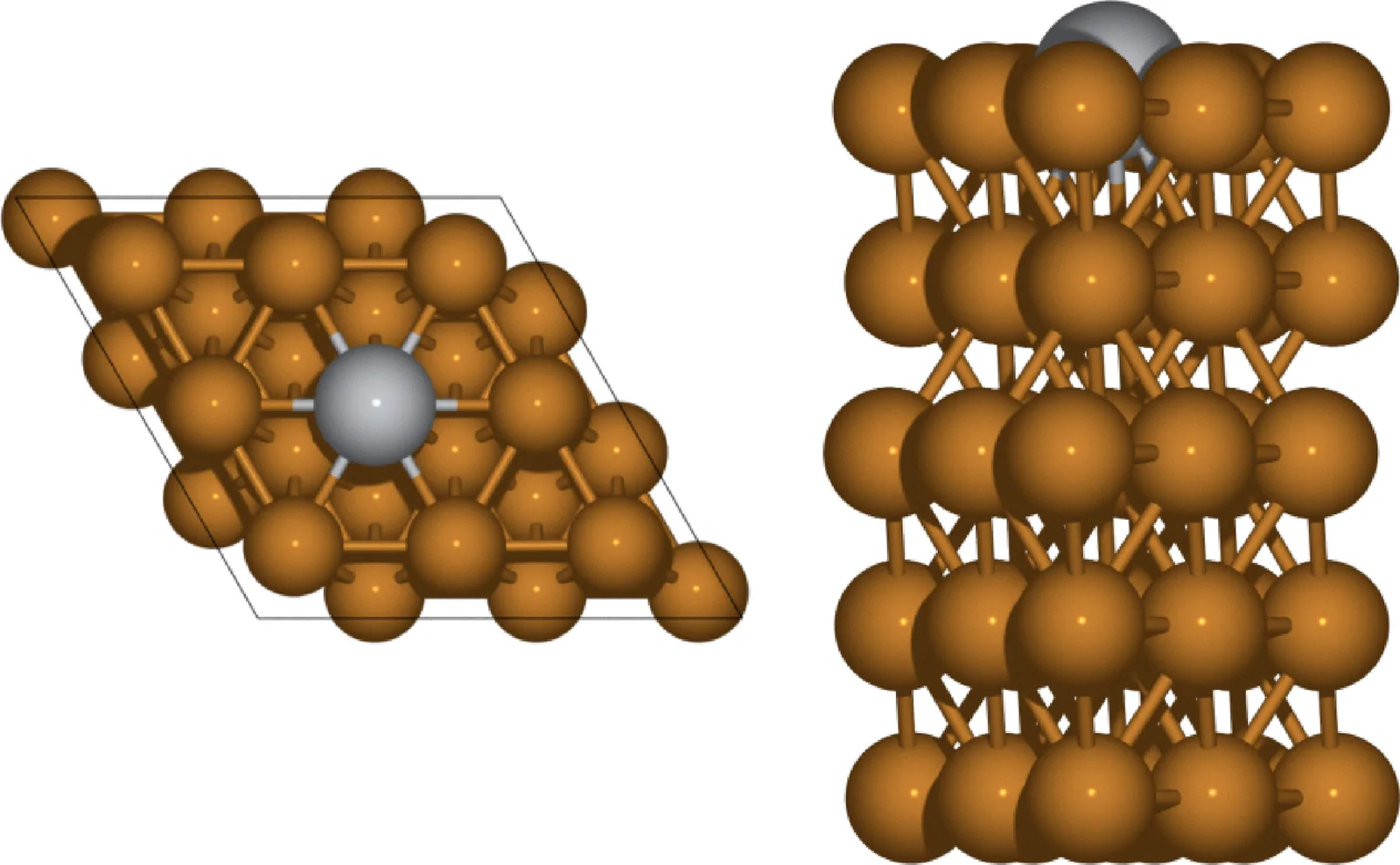
Top (left panel) and lateral (right panel) view of the SAA model employed for the Cu(111)@Ti system.

To model the Cu-based alloys employed in the experiments, we set up two models based on the single-atom alloy (SAA) design. In particular, we substituted a single Cu atom in the surface layer of a Cu(111) structure with either a Ti or Sn atom (Cu(111)@Ti and Cu(111)@Sn, respectively). An example of the resulting structure for the Cu(111)@Ti substrate is shown in Fig. 1. The SAA model was adopted to represent the dilute-doping regime explored experimentally and to isolate the effect of individual Sn or Ti atoms on the catalytic properties of the Cu surface. Experimental characterization does not indicate the pre-*operando* presence of secondary or segregated phases, although the atomic-scale distribution of the dopant atoms cannot be uniquely identified. In this context, the SAA model provides a widely used theoretical framework^30–33^ to probe the local electronic and chemical effects induced by isolated dopant atoms at the surface, which are expected to dominate the catalytic response under reaction conditions. The employed SAA models serve as a pre-*operando* baseline to assess whether the predicted thermodynamic trends remain valid with respect to the stability limits of pure phases, including the possible formation of oxides or intermetallic compounds.

For the two considered SAAs, we increased the system size using a 3 × 3 in-plane supercell with a 6 × 6 × 1 **k**-point mesh grid and an energy cut-off of 50 Ry. The larger in-plane size for the SAAs led to a superficial concentration of the substituent of around 11%, which is comparable with those present in the experimental samples.

The energy cut-off values were obtained through convergence tests performed using several calculations on the bulk structures of the materials considered. The convergence threshold used for the optimisation of the system energies and forces was 10^−4^ Ry and 10^−3^ Ry a.u.^−1^, respectively, for the QE calculations. A vacuum of 20 Å was introduced along the normal direction of the slab surface to avoid interaction with the periodic images. Additional convergence checks were performed on representative slab and adsorbate–surface systems by increasing the plane-wave cut-off, the **k**-point density, and the slab size. These tests showed that the relative adsorption energies changed by less than 1 mRy per atom. For the VASP calculations, the plane-waves energy cutoff was set to 400 eV. The vibrational calculations used to compute zero-point energy and entropic contributions were performed with more stringent electronic convergence criteria, using an electronic energy threshold of 10^−8^ eV for the total energy change.

Initially, we evaluated the reactivity of the substrates under study by computing the surface energy *E*_surf_ as:6
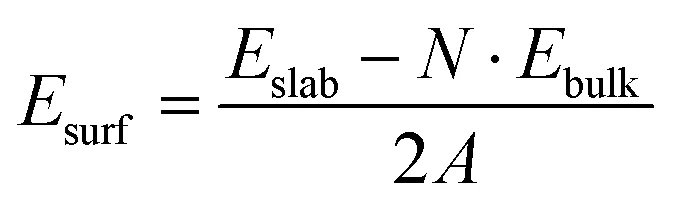
where *E*_slab_ (*E*_bulk_) is the total energy for the slab (bulk) system, *N* is the number of atoms in the slab calculation, and *A* is the in-plane surface area of the slab. Then, to understand whether the atomic substitution of element X on the copper surface was energetically favourable, we computed the exchange energy *E*_exc_ as:

7 *E*_exc_ = *E*^surf^_X@Cu_ + *E*^bulk^_Cu_ − *E*^bulk^_X_ − *E*^surf^_Cu_

where *E*^surf^_X@Cu_ (*E*^surf^_Cu_) is the slab energy with (without) the substitutional atom, and *E*^bulk^_X_ (*E*^bulk^_Cu_) is the energy for the single substituent (copper) atom in its own bulk structure. In this case, a negative value for *E*_exc_ indicates an energetically favourable substitution process.

We considered the adsorption of several molecules and molecular fragments, namely CO_2_, CO, HCOOH, COOH, OCHO, H_2_O, H_2_, H, and OH, that have been identified as key intermediates in the CO_2_RR.^3^ We computed the corresponding adsorption energies as:

8 *E*_ads_ = *E*_surf+mol_ − (*E*_surf_ + *E*_mol_)

where *E*_surf+mol_ is the energy of the adsorbed system, *E*_surf_ is the energy of the isolated slab, and *E*_mol_ is the energy of the isolated molecules or molecular fragments. Also in this case, negative values represented favourable adsorption. For H_2_O and H_2_, we also considered the dissociation process over the substrates under study and computed the dissociative adsorption energies *E*^diss^_ads_ as:

9 *E*^diss^_ads_ = *E*_surf+fragments_ − (*E*_surf_ + *E*_mol_)

where *E*_surf+fragments_ corresponds to the total energy of the system with the fragments adsorbed on the surface.

These molecular adsorption energies of the reaction intermediates over the different substrates are essential to accurately compute the CO_2_RR pathways. To perform this task, it is necessary to explore a vast region of the configurational space to find a global minimum for any adsorbed fragment. We did so by employing Xsorb,^34^ a user-friendly and practical program developed by our computational group that automates the search for the adsorption global minimum. This program, linked to ASE^35^ and Pymatgen^36^ Python libraries, automatically generates a large number of adsorption configurations translating and rotating the molecule relative to the surface. Then, a preliminary optimisation of all the generated structures is performed with relaxed convergence thresholds to identify a sub-set of the most likely adsorption configurations. Finally, these configurations are fully optimized with tighter convergence values to find the global minimum.

Ultimately, we performed climbing-image nudged elastic band (CI-NEB)^37^ calculations to estimate the apparent activation barriers for the key CO_2_RR steps, namely the COOH* and OCHO* formation. CI-NEB calculations were performed using 8 intermediate images and were considered converged when the residual forces acting on the non-fixed images were below 0.05 eV Å^−1^. The transition state was identified from the highest-energy climbing image. Activation energies were computed as the difference between transition and initial state, while free-energy barriers were obtained by adding the corresponding zero point energy and vibrational entropy corrections as explained later in this section.

The kinetics of the Tafel step for the HER on the different structures was also investigated:

10 H* + H* → H_2_(g) + *

where H* denotes the hydrogen atom adsorbed onto the surface of the slab and * denotes the isolated slab. For the CI-NEB calculations of the SAA Cu@Ti structure, the configuration of the adsorbed intermediates was chosen as the most favourable adsorption configuration, whereas the proton adsorption site was chosen as the pristine Cu surface preferred configuration at the first-neighbour distance from the dopant atom. Since the Cu@Sn structure presents an adsorption mechanism governed by the Cu-like surface environments, as shown in the results section, the activation energies are chosen as the ones obtained for the pristine Cu surface.

We employed the computational hydrogen electrode (CHE) model^9^ that allows comparing the chemical potential of a proton-coupled electron transfer step in the reaction to the chemical potential of the hydrogen atom:11
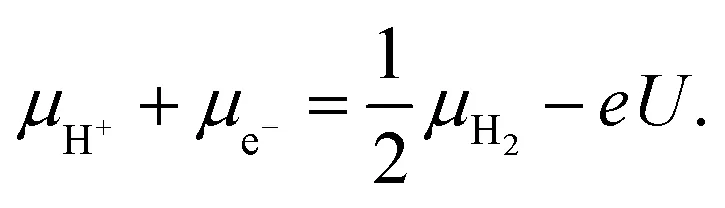


We computed the Gibbs free energy of each reaction step as:^38^12
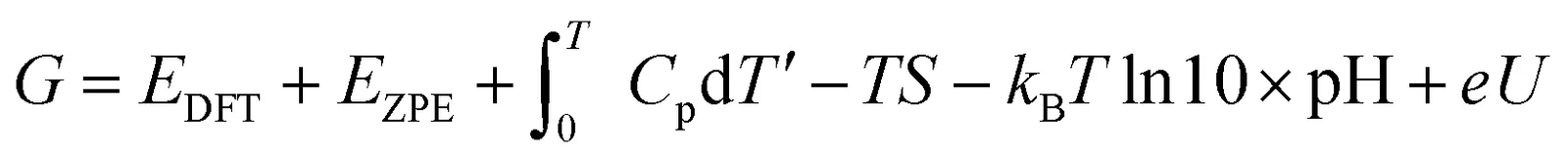
where *E*_DFT_ is the total energy of the system computed through DFT calculations, *E*_ZPE_ is the zero-point energy, *C*_p_ is the heat capacity, *S* is the entropy, *k*_B_*T* ln 10 × pH is the contribution of pH for pH ≠ 0, and *eU* is the contribution of the applied potential for *U* ≠ 0 V *vs.* the standard hydrogen electrode (SHE), which is our reference potential for all our electrochemical reactions. In particular, *E*_ZPE_, *C*_p_ and *S* are calculated from statistical mechanics within the harmonic approximation:13
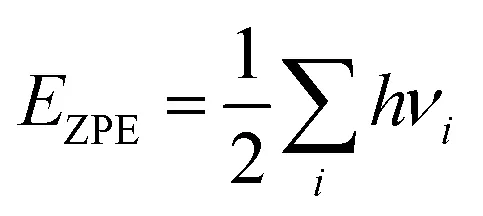
14
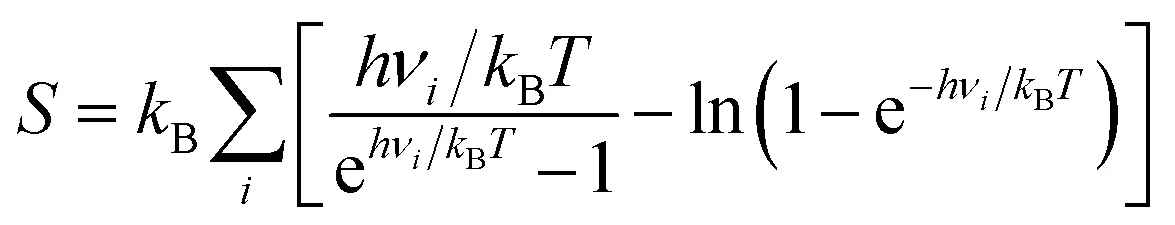
15
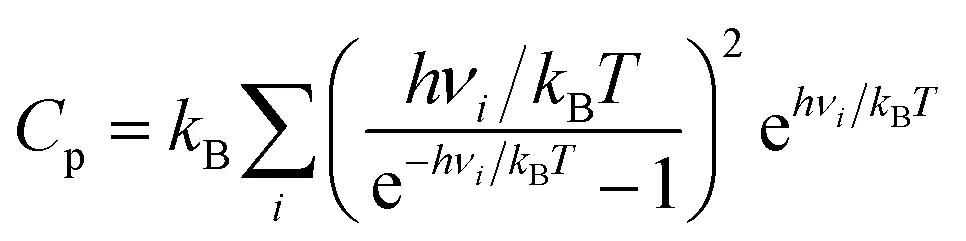
where *ν*_*i*_ are the vibrational frequencies obtained from DFT phonon calculations.^39^ These corrections were calculated with the aid of VASPKIT.^40^ We performed our calculations with pH = 0 and *U* = 0, −0.9 V *vs.* SHE.

Since the activation energies calculated by CI-NEB were built as chemical steps between co-adsorbed species, they are not potential-dependent.^41^ Thus, the applied potential correction −*eU* refers to the preceding H* adsorption (Volmer step).

In the present model, explicit water molecules were not included. This choice was made to retain a simple and internally consistent CHE-based thermodynamic framework for comparing CO_2_RR and HER intermediates across the different catalyst models. The solvent environment, interfacial water structure, and electrochemical double layer were not included either explicitly or implicitly. Accordingly, the calculated free-energy diagrams should be interpreted as comparative thermodynamic descriptors of selectivity, rather than as quantitative electrochemical free energies of the fully electrified solid–liquid interface. This simplified framework was used to identify robust thermodynamic trends arising from the electronic and structural properties of the catalysts, not to describe the full kinetic landscape of the CO_2_RR and HER.

The corrections for the molecules in the gas phase were calculated at *T* = 300 K and pressure *P* = 1 atm. For the H_2_O molecule, *P*_H_2_O_ = 0.035 atm was employed, as this is the saturated vapour pressure at 300 K. The free energy corrections for the adsorbates were calculated at *T* = 300 K, whereas the dependence on pressure and volume can be neglected given the fixed size of the cell. It is important to notice that the adsorption of a single molecule on a surface should not alter significantly the vibrational modes of the slab itself. Therefore, the slab vibrational modes can be neglected in the correction terms of the Gibbs free energy.

## 3 Results

### 3.1 Experimental results

**Fig. 2:**
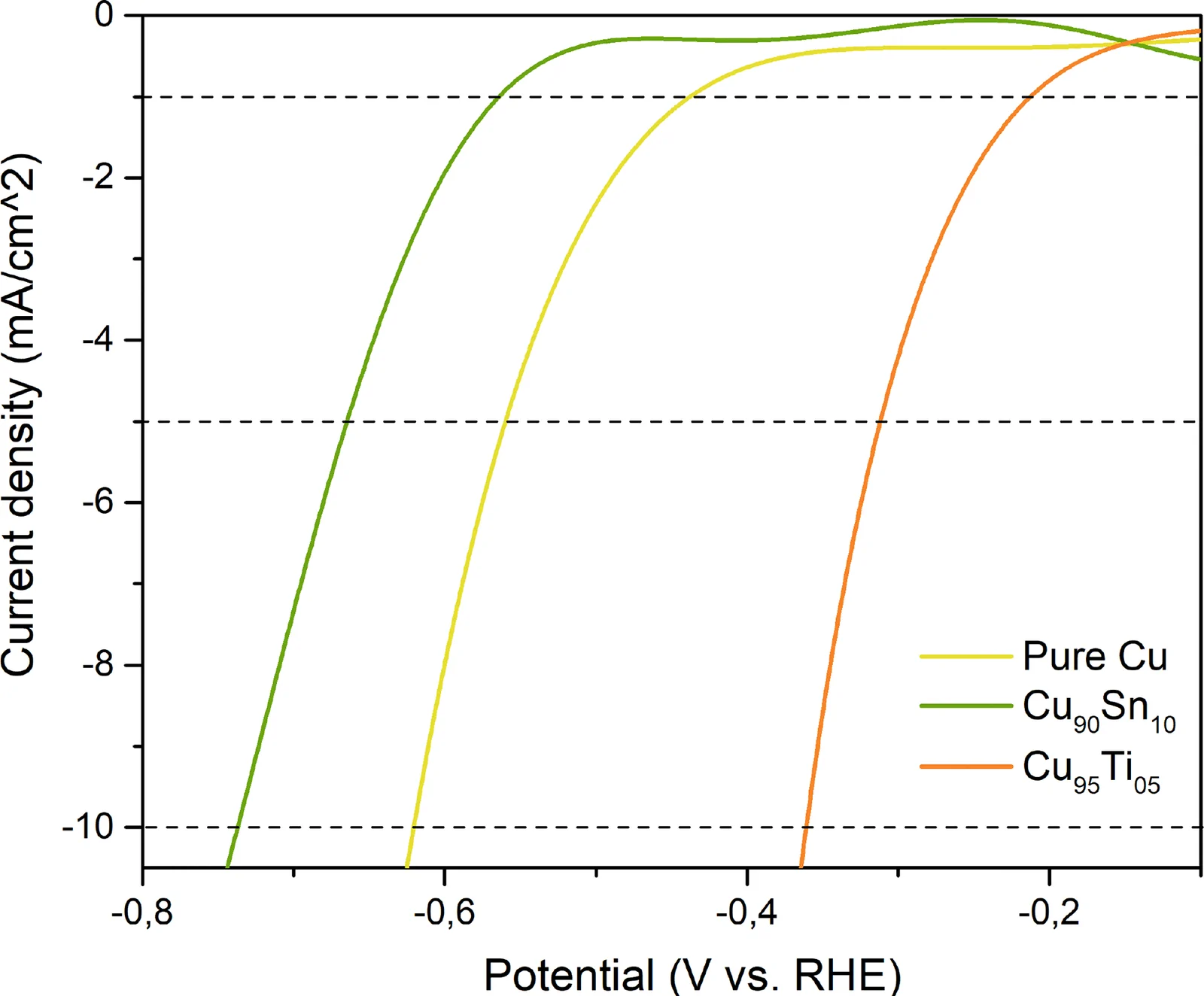
Comparison of the linear sweep voltammetries of pure copper, Cu_95_Ti_05_ and Cu_90_Sn_10_ after the CVs. The three dotted lines for 1, 5, and 10 mA cm^−2^ are highlighted.

A preliminary screening of the electrocatalytic activity of the Cu-based alloys, as a function of the Ti and Sn content, was performed in carbonate buffer. As displayed in Fig. S1 and S2 in the SI, the best performance, in terms of the minimum potential towards cathodic processes, was registered for Cu_95_Ti_05_. Fig. 2 displays linear sweep voltammetry (LSV) carried out after the two cycles of the CVs for these selected samples against the pristine copper one and the potential values are compared with the literature ones in Table S2 in the SI. Herein, Cu@Ti has a significantly lower overpotential at −10 mA cm^−2^ than the pure copper electrode, as expected since the creation of active sites on the surface enhances the HER activity for the optimal value of 1 Ti atom in a 3 × 3 Cu cell.^12^ As for Cu@Sn, a 100 mV higher overpotential specific for that current density can be seen. Nevertheless, the CV curves of the three electrodes behave in agreement with the literature,^12,42^ even though, given the later discussed competition between the HER and CO_2_RR, we cannot express the overpotential in a quantitative fashion. Moreover, the ECSAs of all samples were evaluated and, although the Cu@Sn electrode possesses the highest electrochemically active surface area, its catalytic performance is inferior, demonstrating that HER activity is governed primarily by hydrogen adsorption energetics rather than the surface area. As for the kinetics of the reactions, Tafel slopes were extracted for all electrodes. A slope near 120 mV dec^−1^ is frequently reported for CO_2_ reduction on Cu under reasonably well-controlled hydrodynamics. It falls within the broad distribution of slopes analyzed by Limaye *et al.* in their Bayesian study, which showed that many Cu datasets cluster around non-cardinal values close to 120 mV dec^−1^ rather than the idealized 60 mV dec^−1^.^43^ Ti-modified Cu surfaces tend to weaken CO binding and reduce strong coverage effects. This often produces near-classical slopes (∼110–130 mV dec^−1^), consistent with partially mixed kinetic–transport control but without the large distortions seen in more coverage-sensitive alloys. A value of 117 mV dec^−1^ fits well within this expected regime for the HER.^12^ This value is consistent with the literature since we will see that the Cu@Ti electrode only produces hydrogen. While Sn incorporation strongly shifts selectivity toward *OCHO-mediated formate pathways and suppresses CO adsorption, this typically steepens the Tafel slope relative to pure Cu, but not to the extreme values seen under severe transport limitation. A slope around 110–130 mV dec^−1^ is consistent with Sn-modified Cu systems reported in the literature, where altered intermediate binding and coverage transitions produce moderately inflated slopes.^44^ Our registered value is slightly above this threshold, probably due to the competing HER.

Prior to the electrochemical operations, XRD data of pristine Cu (Fig. S4 in the SI) exhibit a strong reflection related to the (111) peak of the face-centered cubic (FCC) copper structure.^45^ For both alloyed electrodes, a discernible shift in the Cu(111) reflection, nominally located at 19.71° (Mo Kα source), was detected. Although the Cu(200) reflection at 22.72° was not observed for the pure copper sample, both Cu@Ti and Cu@Sn exhibited a similar shift towards lower diffraction angles, with respect to the reference Cu(200) reflection. In the Cu@Ti electrode, the magnitude of this shift increased with the Ti atomic concentration, while no additional diffraction peaks were detected relative to pure copper, indicating the absence of intermetallic phase formation. A more pronounced shift was observed for the Cu@Sn electrode, suggesting a larger lattice expansion. Pure copper exhibits a lattice parameter of approximately 3.58 Å, which increases to 3.61 Å, 3.62 Å, and 3.63 Å for 4%, 6%, and 9.85 at% titanium, respectively. From eqn (2) the calculated strain values are 0.85%, 1.15%, and 1.46%, respectively. The observed linear relationship between the lattice parameter and the copper content is consistent with Vegard's law.^46^ According to this law, the lattice parameter of a solid solution varies linearly with composition, suggesting that Ti or Sn atoms are incorporated into the Cu lattice within the detection limits of XRD, without significant distortions or phase separation. Such behaviour is consistent with partial alloying of the dopant within the Cu lattice. Samples prepared with copper-tin deposition exhibit a similar trend (Fig. S3 in the SI), with FTO peaks and only the copper (111) peak, which is also shifted to lower angles due to the incorporation of the larger tin atoms. The lattice parameter and relative strain were calculated using the same approach as for the titanium samples, yielding a cubic cell parameter of 3.67 Å and a relative strain of 2.63%.

**Fig. 3:**
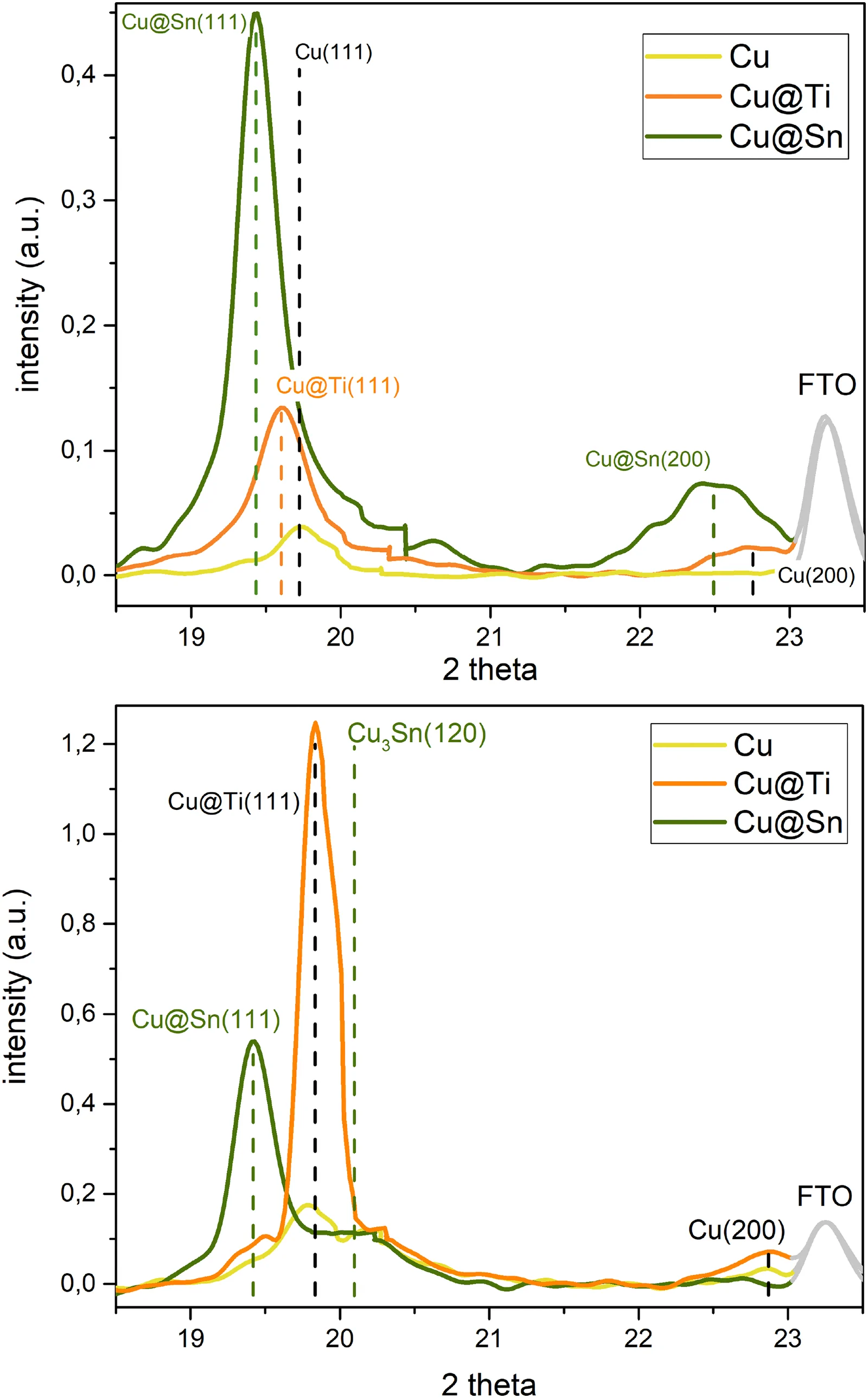
Comparison of XRD data of pre-mortem (up) and post-mortem (down) Cu, Cu@Ti, and Cu@Sn samples at Cu(111) and Cu(200) peak angles (black dotted lines). Cu@Ti is slightly shifted, while the Cu@Sn sample's deviation is significant; moreover, a second peak related to Cu_3_Sn(120) is present in the post-mortem data. The SnO_2_ cassiterite peaks related to the FTO substrate (grey peak) are shown for reference.

*Ex situ* XRD characterization after EC tests of Cu_95_Ti_05_ and Cu_90_Sn_10_ samples was conducted to assess the crystallinity and the formation of intermetallic phases. The characteristic Cu peaks at 19.85° (Cu(111)) and 22.84° (Cu(200)) (Fig. 3) confirm that both the pure Cu and Cu@Ti electrodes retain a dominant (111) texture. In contrast, the Cu@Sn electrode shows a distinct shift in the Cu(111) peak position, accompanied by additional reflections assignable to intermetallic phase formation of Cu_3_Sn(120).^47^ Moreover, while no discernible oxidized phases are visible on the electrode, such species may form transiently under *operando* measurements. In general, post-electrolysis XRD is consistent with the Cu@Sn intermetallic-rich motifs.^48^ Furthermore, the Cu@Ti sample presents a diffraction peak at 16.75° (Fig. S5 in the SI), corresponding to the rutile phase of titanium oxide (TiO_2_), while no peaks associated with Cu compounds were detected, as expected from the phase diagram. Although the equilibrium Ti–H_2_O Pourbaix diagram predicts that TiO_2_ is not the thermodynamically stable phase at the applied cathodic potential (−0.6 V *vs.* RHE at pH 8.3), Pourbaix diagrams do not account for the kinetics of passive-film reduction. Titanium is protected by a highly persistent passive TiO_2_ layer whose reduction in aqueous media is kinetically limited, while the cathodic current under our experimental conditions is predominantly consumed by the hydrogen evolution and CO_2_ reduction reactions. Consequently, the detected rutile phase is more reasonably interpreted as a retained or re-passivated oxide layer rather than evidence of oxide formation under reducing conditions.^49^ Given the difference in atomic radius for Cu and Ti, the shift of the Cu(111) peak suggests the segregation of Ti-rich clusters, possibly related to Ti oxophilicity and, therefore, its proneness to oxidation.

The electrocatalytic activity of the samples was tested in a two-compartment flow cell. The corresponding results are summarized in Table S3 of the SI. As described in Section 2, gas analysis was conducted using the μ-GC by sampling the headspace after 10 minutes of electrolysis, allowing for current stabilization during the chronopotentiometric measurements. Subsequent samples were collected every 10 minutes until the measured peak areas reached a steady state.

**Fig. 4:**
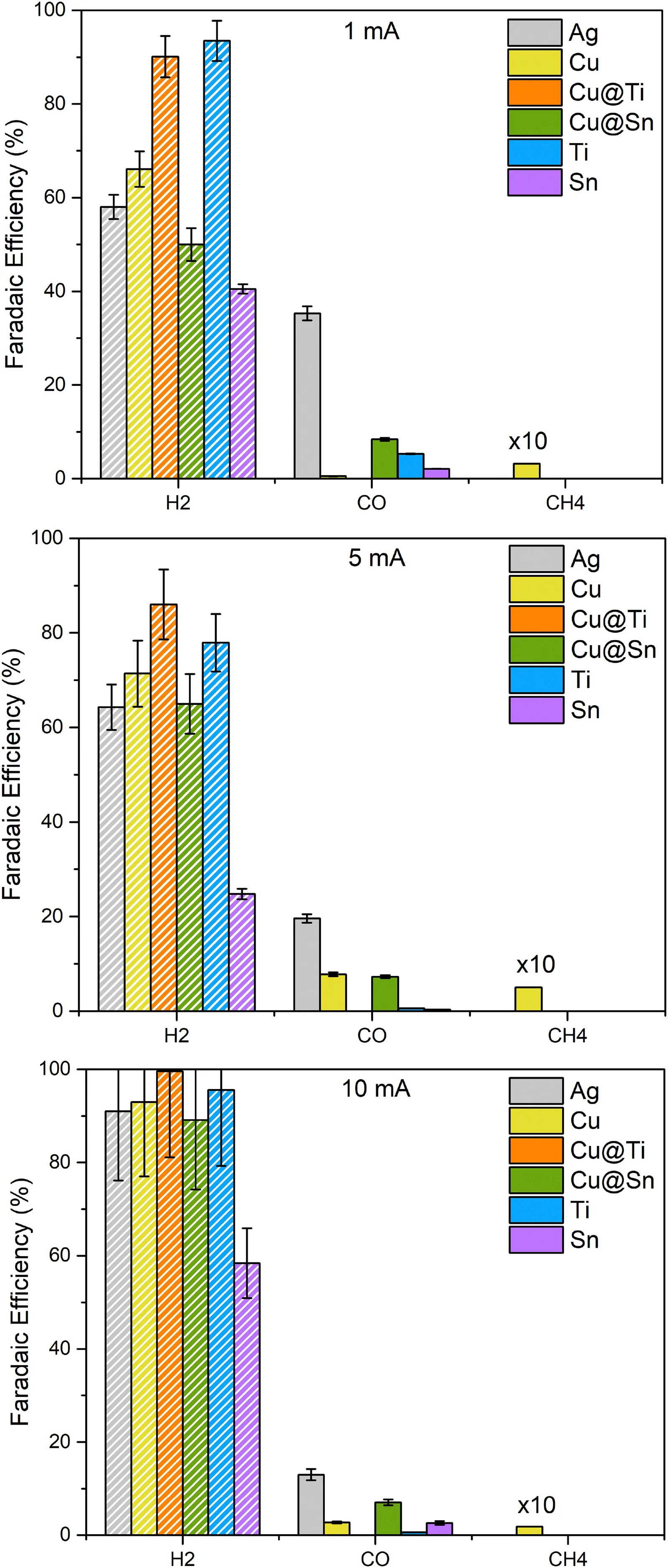
Faradaic efficiencies of H_2_, CO, and CH_4_ for all tested materials at current densities of 1, 5, and 10 mA cm^−2^. The faradaic efficiency of CH_4_ is multiplied by a factor of 10 for clarity in visualization.

The activity of Cu, Cu@Ti, and Cu@Sn samples was compared with that of standard Pt and Ag wires, used as a reference for the calibration of gaseous products. As shown in Tables S3–S5 of the SI, the hydrogen peak areas for all tested electrodes were consistently lower than those obtained with the platinum wire, confirming the validity of platinum as a reference electrode for 100% conversion to hydrogen. Faradaic efficiencies (FEs) for all gaseous products were calculated as the ratio between the moles of each gas produced and the moles of hydrogen produced using the platinum electrode, accounting for the number of electrons exchanged per reaction (*e.g.*, 2e^−^ for H_2_ and CO, 8e^−^ to CH_4_). The measurement performed with a reference Ag wire as WE produces consistent results with the literature in terms of product selectivity, even though the limited faradaic efficiency towards CO points out relatively strong competition by the HER. The results are illustrated for increasing current densities, as shown in Fig. 4, where the uncertainty is obtained with the propagation of the errors related to the gas chromatograph calibration fit.

Although Ag and Cu electrodes are known to facilitate CO and multicarbon product formation, respectively, product analysis revealed that the HER remained the dominant competing reaction across all tested electrodes, particularly at high overpotentials. This suppression of the CO_2_RR could be attributed to a combination of mass transport and chemical effects intrinsic to our flow cell configuration. The limited solubility of CO_2_ in aqueous NaHCO_3_ (∼33 mM at 25 °C) limits the concentration of electroactive CO_2_ at the catalyst surface. These conditions likely limit CO_2_RR activity, particularly for catalysts such as Ag or Cu_90_Sn_10_, which require a stable supply of dissolved CO_2_ for efficient operation.

Moreover, the literature explains how the maximum rate of CO_2_ consumption over the cathode increases inversely with the hydrodynamic boundary layer thickness, as expected for a diffusion-limited process.^50^ In fact, due to our low CO_2_ flow (1 SCCM), the hydrodynamic boundary layer thickness is increased, leading to a higher H_2_ production against CO formation. Another point to realize is that concentration polarization introduces error when data are reported on a RHE scale because the local pH deviates substantially from that in the bulk, in particular at higher current densities (*e.g.* 10 mA cm^−2^), and the surface of a planar cathode would increase steeply towards alkaline pH. These differences can lead to divergent local reaction environments that complicate accurate activity comparisons.

Additionally, the data presented in the tables indicate a pronounced shift in selectivity toward hydrogen at higher applied currents. This trend is consistent with Table S1 in the SI and literature reports,^51^ as increased potentials reduce the energy barrier for the hydrogen evolution reaction, thereby facilitating proton reduction to H_2_.

The silver electrode demonstrated a strong selectivity toward CO production, consistent with literature findings for the applied potential range. Notably, higher CO yields are typically observed within the [−0.7; −1.2] V *vs.* RHE potential range.

No other gaseous products were detected with Ag, and the almost unitary FE suggests the lack of liquid products. Pristine Cu, instead, exhibited a detectable methane signal among the tested materials, as expected. However, the overall selectivity towards carbon-based products was significantly lower than values typically reported in the literature, with the HER largely outcompeting the CO_2_RR with a minimum FE = 65% at 1 mA. This discrepancy may be attributed to the use of sputtered copper, which exhibits different structural and surface properties compared to bulk or electropolished copper. However, the total FE remains well below 100%, suggesting the formation of significant amounts of liquid CO_2_RR products, most likely formate.

In the case of the Cu@Ti electrode, hydrogen was the only detected product, and more than 90% FE is observed under all current conditions, demonstrating a strong shift towards the HER promoted by the inclusion of Ti.

As for the Cu@Sn catalyst, it exhibits a strong shift toward CO production. Indeed, the CO FE approaches 10% at all current values, as expected in our potential range.^16^ The FE for the HER instead reaches a minimum of about 50% at 1 mA. This value is slightly higher than the one obtained for the Cu@Sn alloys at −1.5 V *vs.* Ag/AgCl.^52^ Given the lack of additional gaseous products, this suggests the formation of large amounts of liquid products. Moreover, the presence of parasitic or secondary reactions related to the reduction of SnO_*x*_ species can further lower the overall FE from 100%.

Finally, the faradaic efficiencies obtained for the pure tin and titanium electrodes are consistent with previously reported trends.^53,54^ Titanium exhibits a pronounced selectivity toward the hydrogen evolution reaction, whereas tin predominantly yields liquid-phase products—most likely formate. Furthermore, an increase in applied potential results in a corresponding rise in hydrogen evolution on the tin electrode.

As for the impossibility of reaching 100% of faradaic efficiency at lower currents for both the Cu, Cu@Sn, and Sn samples, liquid products like formate could be present, and in particular, Sn may leach or oxidize, changing local pH and activity and lowering the faradaic efficiencies.^55^

### 3.2 Theoretical results

We started our computational analysis by evaluating the surface energy of the considered metals, considering the most stable surface orientations. As shown in Table 1, Ti(0001) turned out to be the most reactive surface compared to Cu(111) and Sn(111), in agreement with the literature.^56,57^

**Table 1:** Values of the surface energy *E*_surf_ for the different pristine surfaces and values of the exchange energy *E*_exc_ for the two SAA structures

Surface	*E*_surf_ (J m^−2^)	*E*_exc_ (eV)
Cu(111)	1.76	—
Ti(0001)	2.41	—
Sn(111)	0.69	—
Cu(111)@Ti	—	−1.60
Cu(111)@Sn	—	−2.66

When we considered the SAA structures, we found a negative exchange energy *E*_exc_ for both Ti (−1.60 eV) and Sn (−2.66 eV) substitution on the first layer of the Cu(111) surface, indicating an exothermic and favourable process for the creation of these alloys. We also evaluated deeper substitution in the slab, replacing a Cu atom in the second and third topmost layer of the slab. We found a further decrease in energy only for the Ti substitution (*E*_exc_ = −1.73 eV in the second topmost layer and −1.83 eV in the third one). These results suggested that the penetration of the substitutional metals in the first layers of Cu is thermodynamically feasible, but it is important to consider that the present analysis is based on total energies rather than free energies. As a consequence, both the presence of kinetic barriers and the lower entropy associated with the increased ordering of the dopant in the bulk may hinder this process under conditions of limited thermal activation. Therefore, we modelled the SAAs with an atomic substitution in the topmost layer.

Once we decided on the modelling of the experimental substrates, we performed the adsorption energy calculations (Fig. S6 in the SI) of the key intermediates in the CO_2_RR and hydrogen evolution reaction (HER), which is the main competing catalytic process on these substrates. Overall, the adsorption and dissociation analyses on the pristine structures revealed that Ti(0001) binds all CO_2_RR intermediates most strongly, whereas Cu(111) exhibits the weakest interactions. The SAAs generally showed intermediate behavior, with Cu@Ti and Cu@Sn mitigating the excessive reactivity of their dopant metals while preserving moderate adsorption strength. In particular, the adsorption energies on Cu@Sn are rather similar to those on the bare Cu(111) substrate. This can be rationalized by the optimized adsorption geometries, which show that the key intermediates preferentially bind to Cu-rich sites rather than directly to the Sn atom. As a result, the adsorption mechanism is effectively governed by Cu-like surface environments. Previous studies have shown that the effect of Sn dopants becomes more pronounced when more reactive surfaces, such as stepped sites, are considered, when the dopant coverage is increased,^58^ and when explicit solvation is included in the model.^59^ Under such conditions, the sensitivity of adsorption energetics to the local chemical environment around Sn can be enhanced, leading to more significant differences in the corresponding free energy diagrams.

We also performed Bader charge analysis using the code developed by Henkelman and co-workers,^60–63^ which provides insight into the weak H adsorption on the ideal metallic Cu@Ti SAA model. In the pristine alloy, the Ti dopant donates approximately +1.25*e* to neighbouring Cu atoms, leading to a localized redistribution of charge. Upon H adsorption, this balance is perturbed: the nearby Cu atoms lose part of their acquired negative charge, while the Ti atom remains positively charged. The resulting local electrostatic environment weakens the stabilization of H, explaining the positive Δ*G*(H) obtained for the Volmer descriptor on Cu@Ti.

This positive Δ*G*(H) should not be interpreted as evidence that the experimental Cu@Ti electrode is intrinsically inactive toward the HER. Instead, it shows that isolated Ti atoms embedded in a Cu-like metallic surface do not strongly stabilize H in the initial Volmer step. The Tafel-step calculations provide complementary information: once H is present, H–H recombination/desorption on Cu@Ti proceeds through a finite but relatively moderate activation barrier within the present unsolvated model. Thus, while H formation is thermodynamically less favourable on the ideal Cu@Ti SAA, subsequent H_2_ formation is not strongly kinetically hindered. Together with the experimentally observed evolution of Cu@Ti toward TiO_2_/TiO_*x*_-rich motifs, these results reconcile the CHE Volmer descriptor with the strong HER selectivity measured experimentally.

The reduced H_2_ and H_2_O dissociation energies of the SAA models compared with pristine Ti further indicate that the ideal SAA structures should be regarded as pre-*operando* electronic baselines rather than fully representative HER-active Ti-rich surfaces.

**Fig. 5:**
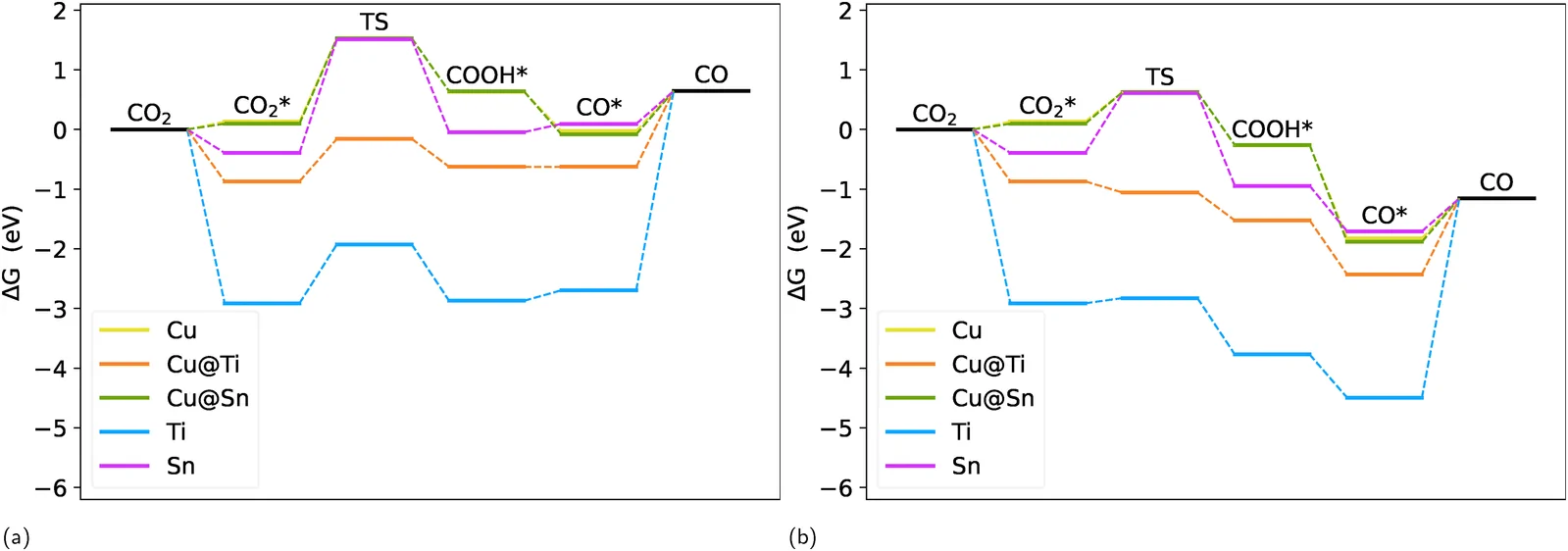
Free energy diagram of the CO_2_RR to CO at 0 V *vs.* SHE (panel (a)) and at −0.9 V *vs.* SHE (panel (b)) for the different surfaces.

**Fig. 6:**
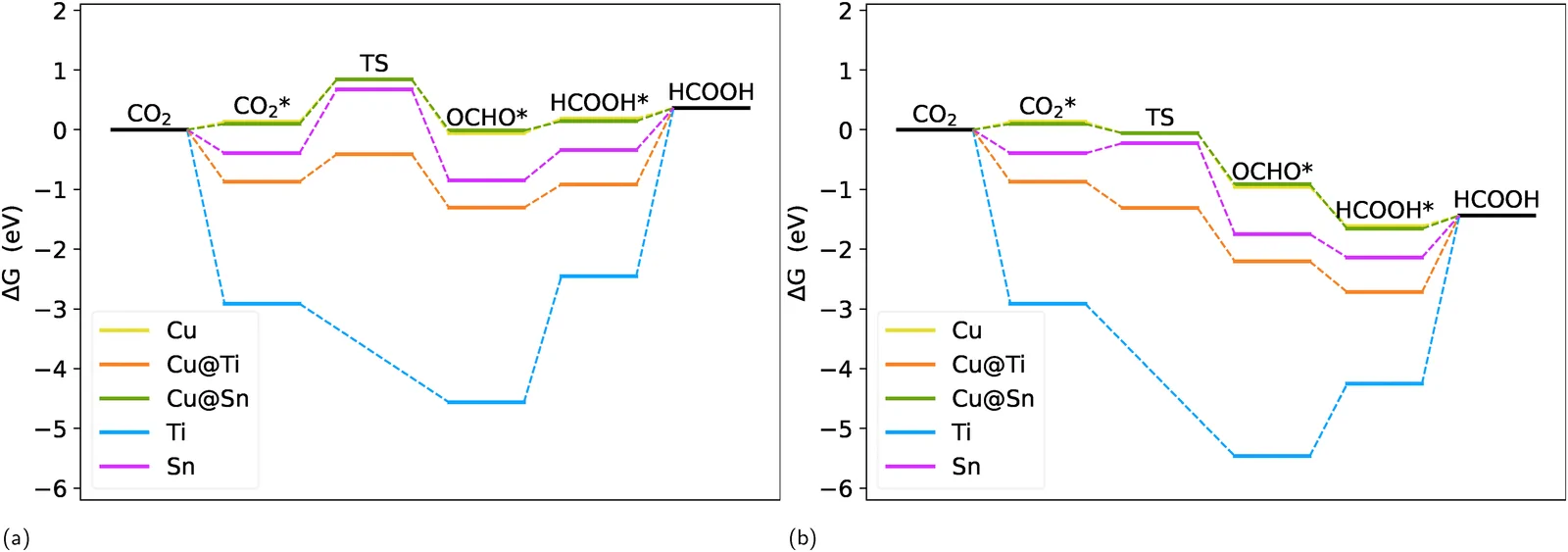
Free energy diagram of the CO_2_RR to HCOOH at 0 V *vs.* SHE (panel (a)) and at −0.9 V *vs.* SHE (panel (b)) for the different surfaces.

**Fig. 7:**
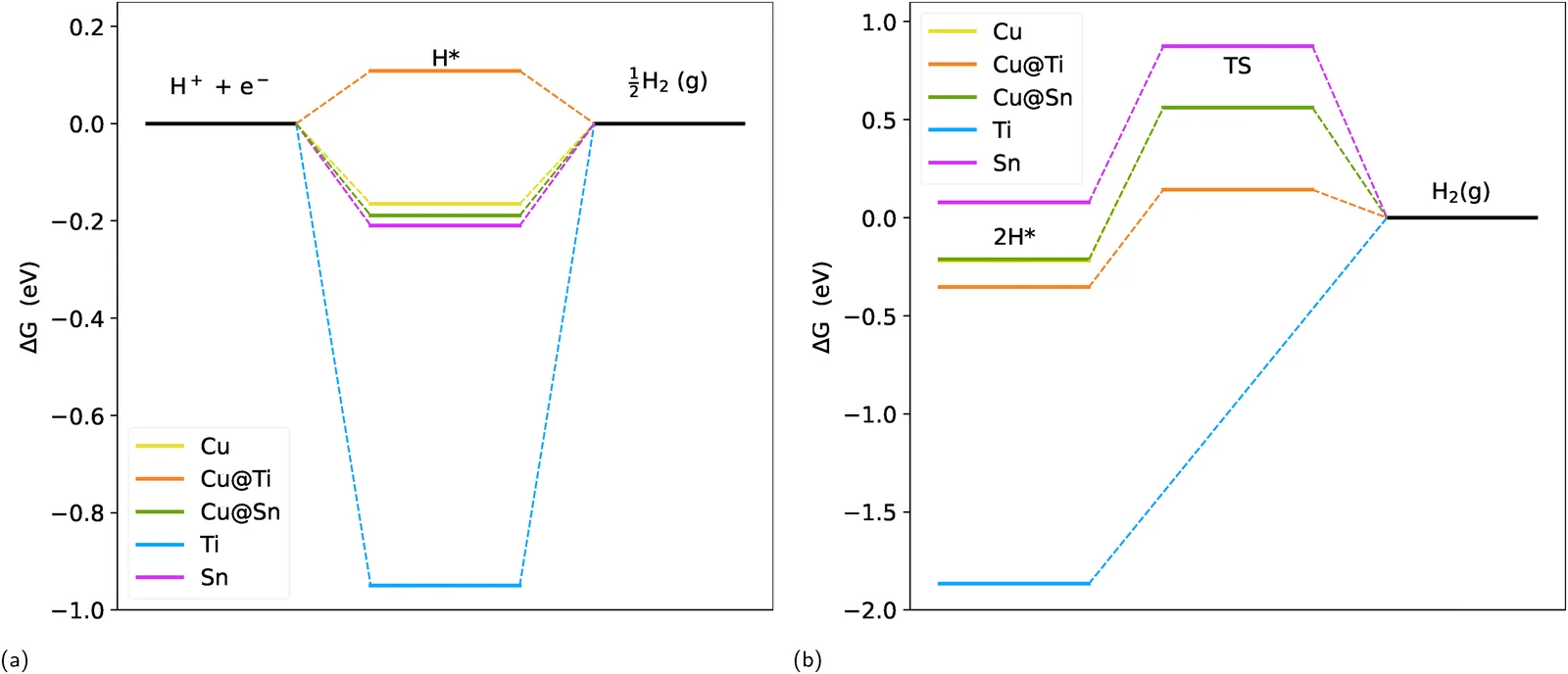
(Panel (a)) Free energy diagram of the HER for the different surfaces. (Panel (b)) Free energy diagram of the Tafel step for the surfaces that present an activation energy.

The optimised adsorption configurations were used to compute the Gibbs free energy according to eqn (12) (complete data are reported in Section IV of the SI), from which the free energy diagrams were constructed for CO_2_ reduction to CO (Fig. 5a) and to formic acid (Fig. 6a). The potential-dependent pathways for *U* = −0.9 V *vs.* SHE are presented in (Fig. 5b and 6b), respectively. The corresponding HER pathways are shown in Fig. 7a, along with the Tafel step activation energy shown in Fig. 7b. The potential-dependent HER diagram can be found in Fig. S7 in the SI. These diagrams illustrate the evolution of the Gibbs free energy along the reaction coordinates and enable a direct comparison of the relative selectivity of the different surfaces toward competing pathways. Among the investigated systems, pristine Ti(0001) exhibits high energy gains for all intermediates, suggesting a tendency toward overbinding, surface oxidation, and possible catalyst poisoning. In particular, for this structure, no activation barrier was found for the HER Tafel step and for the reduction of CO_2_ to the OCHO* intermediate.

For the remaining surfaces, the trends observed in the adsorption analysis are largely preserved, with SAA structures displaying intermediate free energy values between those of the corresponding pristine phases. The CO_2_RR diagrams show that the *OCHO formation is thermodynamically more favourable than COOH* formation across all surfaces, with increasing Sn or Ti content further amplifying this preference. At the same time, higher dopant content enhances the energy difference between COOH* and CO*, as well as between the OCHO* and the HCOOH* step, indicating a progressive modulation of the reaction energetics. Notably, Cu@Sn closely resembles pristine Cu(111) in its free energy profile, whereas Cu@Ti retains the strong binding character of Ti(0001), albeit in attenuated form. Finally, Cu@Ti is the only system exhibiting a positive free energy for the HER adsorption step, whereas the co-adsorption of two H atoms on the surface is favourable compared to the molecule in the gas phase. This energy difference between Volmer and Tafel steps can be explained by considering the mutual interaction between the two adsorbed H* species and the doped surface, leading to a more stable co-adsorbed state from the isolated H* adsorption energy. The presence of a Cu-like adsorption site contributes to the stabilization of the co-adsorbed configuration.

From the CI-NEB activation-energy profiles for the CO* pathway, calculated by CI-NEB, we identified COOH* formation as the potential-determining step for all systems. The formation of OCHO* in the HCOOH pathway presents a lower activation barrier than COOH* formation. The computed free energy diagrams align well with the previous literature, confirming the strong tendency of bare copper towards the HER in acidic media^64^ and the most probable reaction pathway for the CO_2_RR to CO and HCOOH.^31^ Our calculations for pure titanium substrates further support that this material predominantly reduces water.^65,66^ For the considered SAAs, the results for the Cu@Ti model show good agreement with a similar study, where a Ti atom was adsorbed onto the surface rather than substituted,^31^ whereas for Sn systems, prior studies have reported potential-dependent thermodynamics and free-energy pathways on pure Sn (metal/oxides) and on Cu–Sn interfaces or surface-alloys.^58,67,68^

**Fig. 8:**
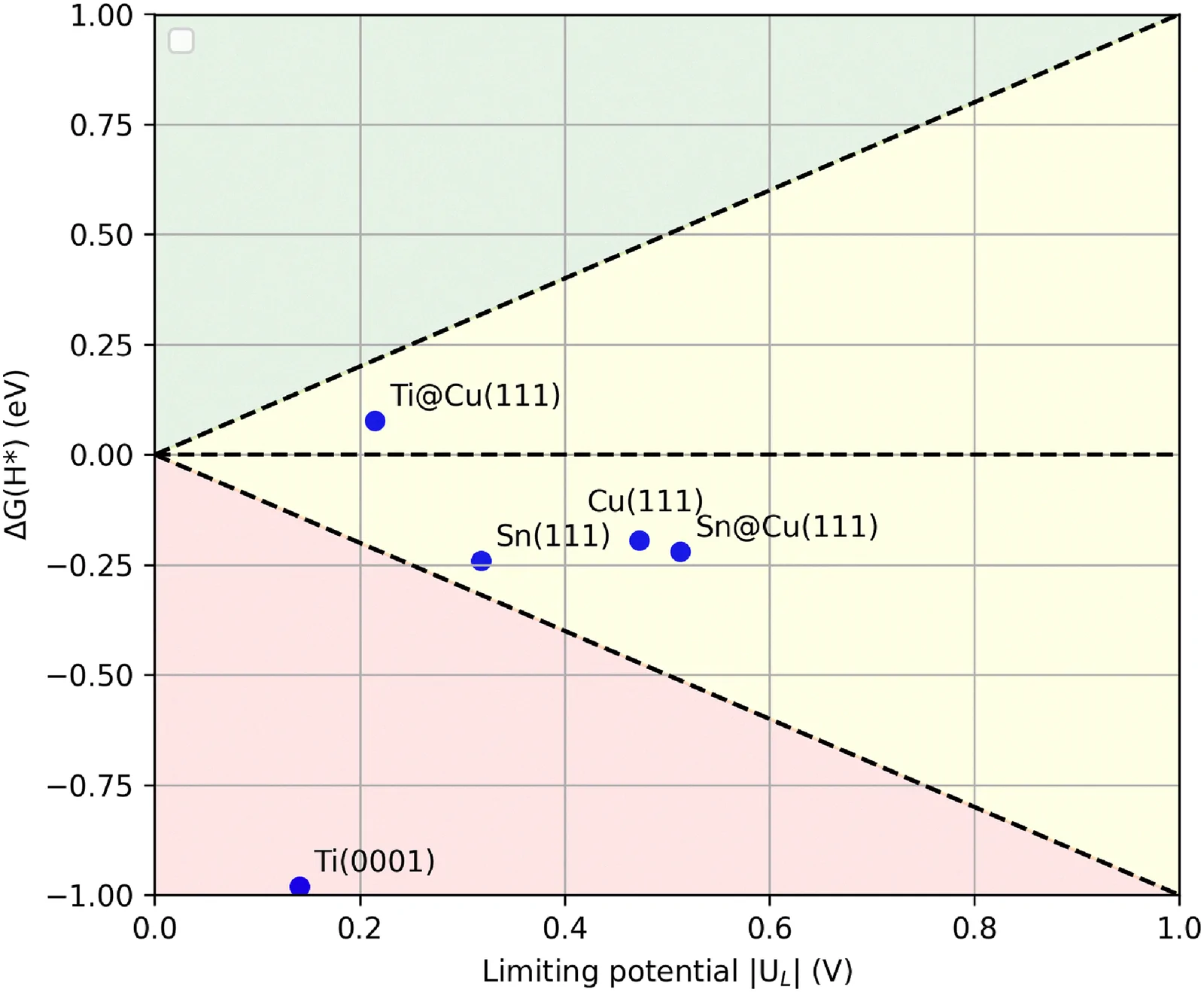
Relationship between the absolute value of limiting potential for the CO_2_RR to CO and the Gibbs free energy of H*. Green region: Δ*G*(H*) > 0 and |*U*_L_| < |Δ*G*(H*)|. Yellow region: |*U*_L_| > |Δ*G*(H*)|. Red region: Δ*G*(H*) < 0 and |*U*_L_| < |Δ*G*(H*)|.

To rationalize the experimental results and elucidate the origin of the observed selectivity, we related the computed Gibbs free energy of adsorption for the HER, Δ*G*(H*) to the CO_2_RR limiting potential |*U*_L_| for the CO pathway, defined as the energy of the potential-limiting step, mentioned previously, divided by the elementary charge. |*U*_L_| represents the minimum potential required for the reaction to be exothermic. This analysis can help to visualise how different substrates promote either the HER or CO_2_RR to CO. As shown in Fig. 8, Ti(0001) is located in the red region, which indicates a high likelihood of H poisoning for the surface: the application of a potential *U* would further strengthen the adsorption of protons on the surface before the onset of the CO_2_RR. Cu, Sn, and Cu@Sn are located in the negative Δ*G*(H*) part of the yellow region, which is the area where the substrates could support both the HER and CO_2_RR with selectivity towards the former.^69,70^ Finally, Cu@Ti is the only system located in the upper part of the yellow region, which indicates selectivity towards the CO_2_RR.^71^ However, Cu@Ti and Ti(0001) results need to be contextualized and related to the strong adsorption and dissociation values for O-binding intermediates that would promote oxidation and possible poisoning of the considered structures, experimentally evolving toward TiO_2_-rich, HER-active motifs. In particular, the absence of a Tafel barrier for Ti within the explored NEB pathway suggests that once H is adsorbed on Ti-rich motifs, H_2_ formation is not kinetically hindered in the same way as on Cu-like surfaces. This behaviour indicates a strong tendency of Ti-containing surfaces to stabilize adsorbed hydrogen, which may lead to high H coverage and possible H poisoning. In this sense, the NEB results are consistent with the strong HER activity observed experimentally.

## 4 Discussion

The experimental and theoretical results collectively highlight the complex interplay between surface composition, morphology, and reaction environment in determining CO_2_ reduction selectivity on Cu-based electrodes modified with Ti or Sn. Although the sputtered films share similar microstructural features and roughness, their electrochemical behavior diverges sharply, emphasizing the sensitivity of CO_2_RR pathways to surface chemistry and phase evolution during operation.

For the Cu@Ti films, the faradaic efficiency was dominated by hydrogen evolution (>90%), with negligible CO or other gaseous CO_2_RR products. Post-mortem XRD revealed the formation of rutile TiO_2_, indicating that Ti readily oxidizes and segregates under reaction conditions. These observations suggest that the catalytically active surface during electrolysis is TiO_*x*_-rich rather than a metallic Ti–Cu interface. These oxide domains may partially reduce under cathodic potentials, exposing metallic Ti or sub-oxides (TiO_*x*_), which are active for the HER and poor catalysts for the CO_2_RR. Titanium surfaces exhibit moderate hydrogen binding energies, especially after surface oxide removal, which makes titanium good at adsorbing protons and converting them into H_2_ gas. It also has a relatively low work function, which helps in electron transfer to protons at the surface and promotes the Volmer step, a key part of the HER. We therefore attribute the measured behavior primarily to the HER on TiO_*x*_ domains, while the underlying Cu likely plays a limited role under the tested conditions. *Operando* surface analysis will be necessary to confirm this hypothesis, but this confirms that the SAA is only a pre-*operando* electronic baseline; once Ti oxidizes, the experimentally relevant surface changes the regime. Our DFT calculations are consistent with this overall picture, as the bare Ti system exhibits a pronounced preference for hydrogen stabilization that results in a HER-biased landscape. The HER behaviour of Cu@Ti should be interpreted by considering both the ideal SAA descriptor and the experimentally observed surface evolution. Although H* formation on the ideal metallic Cu@Ti SAA is thermodynamically less favourable, the Tafel-step calculations indicate that H–H recombination/desorption is not strongly kinetically hindered once H* is present. Moreover, the strong stabilization of OH* and the large negative dissociative adsorption energy of H_2_O on Cu@Ti, around −4 eV as shown in Fig. S6 in the SI, point to a pronounced tendency of Ti-containing sites to activate oxygenated species. This is consistent with the formation of TiO_2_ observed experimentally and suggests that the reconstruction toward TiO_2_/TiO_*x*_-rich motifs further drives the catalysts toward HER-dominated behaviour.

In contrast, Cu@Sn exhibited a modest CO faradaic efficiency (∼10%) and significant unaccounted charge, implying the formation of liquid products. The post-electrolysis XRD pattern showed a reflection corresponding to Cu_3_Sn, consistent with surface Sn enrichment. A pronounced shift of the Cu(111) and Cu(200) reflections was also observed (Fig. 3), indicative of lattice expansion resulting from the incorporation of Sn into the Cu lattice. The additional peak at 43.54°, attributed to the Cu_3_Sn(120) phase,^72^ suggests that local Sn enrichment occurs through selective dissolution and re-deposition during electrochemical operation. This process likely induces compositional inhomogeneities as Sn becomes locally concentrated and reacts with Cu to form Cu_3_Sn. Prior studies^16^ have correlated the formation of Cu–Sn intermetallic phases with a shift in CO_2_RR selectivity towards CO and formate production at higher Sn contents (>20 at%), suggesting that our system may follow a similar trend. Although liquid product analysis was not performed, the missing faradaic efficiency is most plausibly attributable to formate formation. Direct quantification by NMR or ion chromatography will be required to confirm this assumption and to assess the true selectivity of the Cu–Sn surface. This shift in selectivity was previously explained with a shift in adsorption preference from the C-bound *COOH intermediate to the O-bound *OCHO intermediate, due to the charge redistributing from Sn to Cu, which creates regions of localized positive charge on the Sn sites. Therefore, with increasing tin content, these localized positive sites hinder nucleophilic attack of the CO_2_ carbon, making *COOH adsorption (and the CO pathway) less favourable.^16^ The theoretical simulations are consistent with this picture, showing that the energetics of the Cu@Sn system align with those of the pristine Cu. While under more reducing conditions, the selectivity is known to shift toward liquid products. Although these products could not be detected experimentally, our DFT calculations (Fig. 6) indicate a clear preference for OCHO formation on the Sn substrate, in line with previous experimental reports.^66,73^ The observed modest CO faradaic efficiency could also derive from the onset of activation barriers towards CH_4_ formation on the Cu@Sn system.

The DFT analysis provides complementary insight into the catalytic behaviour of the investigated systems within the theoretical framework introduced above. To rationalize dopant-dependent selectivity, we employed SAA models to isolate the intrinsic ligand effects of dilute Ti and Sn atoms, complemented by pristine Cu(111), Ti(0001), and Sn(111) surfaces to bound the reactivity space. Although these models represent idealized boundary conditions—isolated dopants embedded in Cu(111) under simplified electrochemical conditions (such as pH = 0), the resulting trends are remarkably consistent with both literature reports and our experimental observations. In particular, the computed behaviour of the pure surfaces defines the limiting reactivity patterns expected for Cu-, Ti-, and Sn-rich environments, while the SAA structures capture how local electronic perturbations induced by isolated dopants modulate key CO_2_RR intermediates. This modulation rationalizes the experimentally observed differences in selectivity. Overall, the agreement between theory and experiment emerges when the computational models are interpreted as physically motivated boundary systems that capture the relevant compositional and electronic features of the catalysts, despite their structural and dynamic complexity under reaction conditions. Because solvent, interfacial water, and double-layer effects are not included in the present model, the CHE profiles and CI-NEB barriers should be interpreted as comparative descriptors within a consistent simplified framework, rather than as quantitative electrochemical free energies or kinetic barriers.

Within these limits, our Δ*G*-based descriptors, especially Δ*G*(H*)^‡^ and Δ*G*(COOH*)^‡^, and the identification of *COOH formation as the uphill step on Cu-rich sites are consistent with established Cu mechanistic models, where *COOH formation and *CO stabilization govern CO_2_RR selectivity.^51^ The associated kinetic signatures, including the Tafel behaviour tied to this first proton–electron transfer step, are likewise consistent with the Cu literature.^51^ For Cu–Sn, the emergence of O-bound *OCHO on Sn-containing environments, while *COOH remains uphill on Cu-rich motifs, aligns with established mechanisms in which Sn promotes CO/formate pathways through local electronic effects at Cu–Sn interfaces. Consistent with this picture, single-atom-alloy studies and flow-cell measurements likewise attribute the CO/formate bias to the coordination environment around isolated Sn atoms on Cu.^58^ For Ti/TiO_2_, the strong stabilization of H* and the resulting HER biased landscape on Ti-rich motifs are consistent with broader evidence that Ti/TiO_2_ surfaces favour water reduction and exhibit intrinsically poor CO_2_RR activity in aqueous media, in line with previous electrocatalytic and photocatalytic studies.^74^ Our study adds a mechanistic perspective that explicitly incorporates phase evolution observed in post-electrolysis XRD: TiO_2_ formation on Cu@Ti and Cu_3_Sn development on Cu@Sn redefine the position of the active surface in descriptor space. Complementarily, our coverage-dependent selectivity map identifies when Cu-like SAA motifs retain *COOH-limited behaviour and when Sn-enriched intermetallic environments favour O-bound pathways, thereby explaining the transition between H_2_, CO, and formate across different compositions and potentials. Together, these results yield a phase-evolution-aware, coverage-sensitive mechanism with clear transferability.

Our results define a clear stability–selectivity design principle for doped-Cu CO_2_RR electrocatalysts. Ti-containing reconstructed or TiO_*x*_-rich motifs strongly stabilize hydrogen-related intermediates and drive the surface into the HER-favoured regime. By contrast, post-transition dopants such as Sn remain redox-stable and form Cu–Sn intermetallics (Cu_3_Sn) that stabilize O-bound intermediates (notably *OCHO), thereby promoting CO/formate pathways. Mechanistically, these trends map onto a descriptor space defined by Δ*G*(H*)*^‡^ and Δ*G*(COOH*)*^‡^ (with *OCHO on Sn sites): Ti-derived oxides push the catalyst toward *H-favoured adsorption, whereas Sn-enriched ensembles lower the barrier for O-bound routes. This yields a general rule: retain metallic/intermetallic dopants to access O-bound CO_2_RR pathways and avoid oxophilic dopants that reconstruct into HER-active oxides. Supported by the observed phase evolution from TiO_2_ formation on Cu@Ti to Cu_3_Sn development on Cu@Sn, this stability-aware, descriptor-based filter offers a transferable guideline for exploring the broader Cu–M design space.

Finally, mass-transport effects and CO_2_ availability likely contributed to the overall low CO_2_RR selectivity across all samples. The CO_2_ flow rate (1 SCCM) and the cell configuration used here inherently limit reactant concentration near the electrode. Under such conditions, hydrogen evolution competes strongly, particularly on oxide-rich or less conductive surfaces. Higher CO_2_ fluxes, gas-diffusion electrodes, or pressurized cells would allow a more intrinsic comparison of catalytic performance.

## Conclusions

This study shows that the redox stability of the dopant is a primary factor governing CO_2_ reduction selectivity on Cu. Under operating conditions, Ti migrates and oxidizes to TiO_2_, which shifts the surface toward hydrogen evolution, whereas Sn enriches into Cu–Sn intermetallic motifs that stabilise O-bound intermediates and steer products toward CO/formate. In our composition-spread thin-film libraries prepared by magnetron co-sputtering, this manifests as negligible CO_2_ reduction on Cu@Ti and a modest yet reproducible CO signal (10%) on Cu@Sn, alongside signatures consistent with liquid-product formation. A simple mechanistic picture emerges from the combined DFT–CHE and CI-NEB calculations and analysis: hydrogen binding strength and the apparent barrier for COOH/OCHO formation together organise the branching between the HER and CO/formate formation on Cu-based surfaces. Single-atom-alloy models of Cu@Ti and Cu@Sn capture the ligand effects on Cu-rich sites, and, when interpreted together with the post-electrolysis phases (TiO_2_ on Cu@Ti; Cu_3_Sn on Cu@Sn), point to a simple stability–selectivity rule: metallic or intermetallic dopants should be maintained under bias to enable O-bound CO_2_-reduction pathways, whereas oxophilic dopants that oxidize and reconstruct will instead drive the catalyst into HER-active oxide states. This rule is transferable across the broader Cu–M design space.

Future work should combine *operando* characterization (XPS, Raman, and XAS) with quantitative liquid-product analysis to confirm active species and product distributions, and extend testing to gas-diffusion geometries where CO_2_ availability is not limiting. The theoretical framework developed here could also be expanded to explicitly include oxidized interfaces and potential-dependent solvation, enabling a more realistic link between the model and the experiment. Ultimately, the synergy between controlled sputter-based synthesis, *operando* diagnostics, and computational modeling offers a robust route to disentangle the roles of subsurface alloying and dynamic surface reconstruction in shaping CO_2_RR selectivity on Cu-based catalysts.

## Author contributions

Francesco Barberini: formal analysis, investigation, writing – original draft. Antonio Barile: formal analysis, investigation, writing – original draft. Paolo Restuccia: methodology, writing – review & editing. Pierfrancesco Atanasio: validation, writing – review & editing. Daniele Passeri: validation, writing – review & editing. Devis Di Tommaso: validation, writing – review & editing. Raffaello Mazzaro: conceptualization, funding acquisition, supervision, writing – review & editing. Luca Pasquini: supervision, writing – review & editing. M. Clelia Righi: conceptualization, funding acquisition, supervision, writing – review & editing.

## Conflicts of interest

There are no conflicts to declare.

## Supplementary Material

CP-028-D6CP01600F-s001

## Data Availability

The data supporting this article have been included as part of the supplementary information (SI). The supplementary information contains additional experimental data, including electrochemical measurements, XRD characterization and gas chromatography results, together with DFT adsorption and dissociation energies, HER free-energy data, and calculated total-energy and free-energy contributions. See DOI: https://doi.org/10.1039/d6cp01600f. The in-house Xsorb software employed for computing molecular adsorption energies can be found at https://gitlab.com/triboteam/xsorbed, published with DOI https://10.1016/j.cpc.2023.108827.
