# Supplementary material for: Dopant redox stability as a design principle for selective CO2 electroreduction on Cu
Source: Phys Chem Chem Phys. 2026 Jul 30;28(35):21230–45. doi: 10.1039/d6cp01600f (PMC13487734; doi:10.1039/d6cp01600f)
Supplement: Supplementary file 1 [file CP-028-D6CP01600F-s001.pdf]

Supplementary Information (SI) for Physical Chemistry Chemical Physics.
This journal is © the Owner Societies 2026

Supplementary Information to: "Dopant Redox Stability as a Design Principle for Selective CO_2 Electroreduction on Cu"

Francesco Barberini,[1,*] Antonio Barile,[1,*] Paolo Restuccia,[1,†] Pierfrancesco Atanasio,[2] Daniele Passeri,[2] Devis Di Tommaso,[3] Raffaello Mazzaro,[1,‡] Luca Pasquini,[1] and M. Clelia Righi[1]

[1] *Department of Physics and Astronomy, University of Bologna, Via Carlo Berti Pichat 6/2, Bologna (Italy)*
[2] *Department of Basic and Applied Sciences for Engineering, Sapienza University of Rome, Via A. Scarpa 14, 00161, Rome (Italy)*
[3] *School of Physical and Chemical Sciences, Queen Mary University of London, Mile End Road 327, London E1 4NS (UK)*

I. EXPERIMENTAL DATA

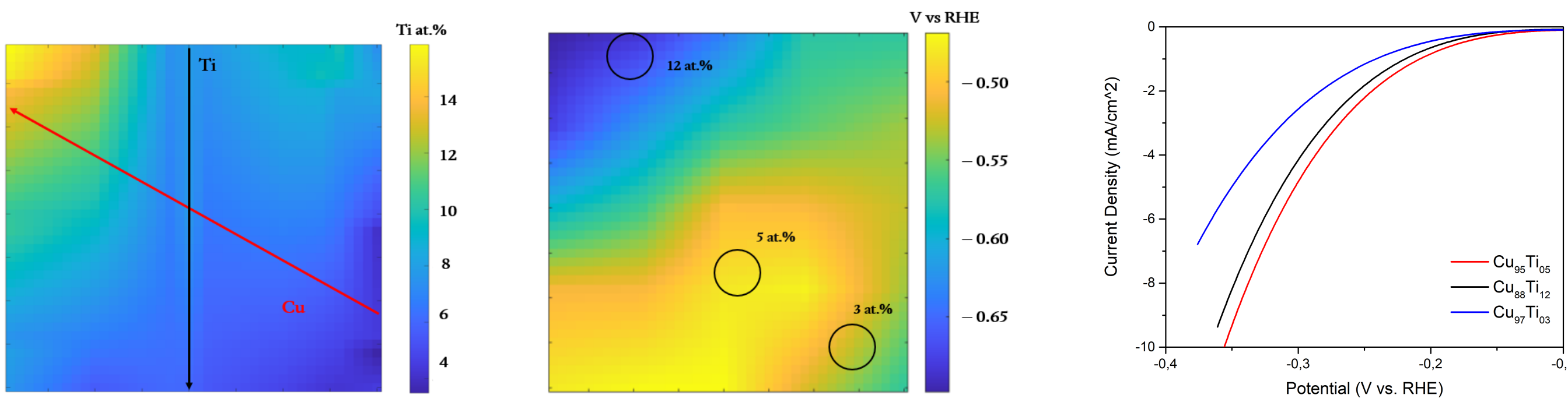

(a) (b) (c)

FIG. S1: Copper-Titanium gradient concentration (left panel) and overpotential at - 10 mA/cm^2 (middle panel). Comparison of cyclovoltammetries (right panel) of different Cu@Ti compositions. $Cu_{95}Ti_{05}$ shows the lowest overpotential.

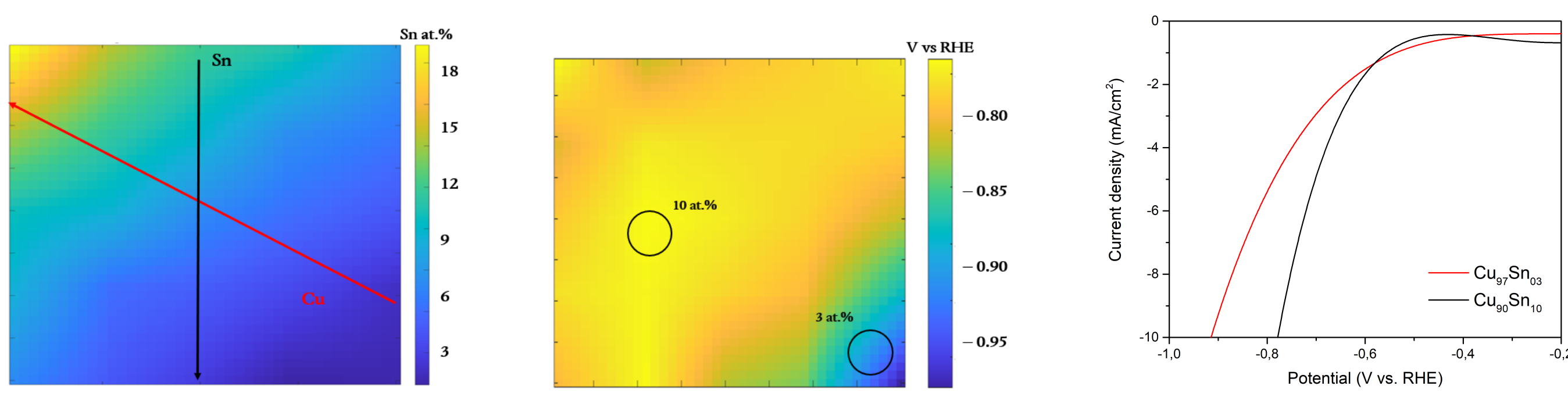

(a) (b) (c)

FIG. S2: Copper-Tin gradient concentration (left panel) and overpotential at - 10 mA/cm^2 (middle panel). Comparison of cyclovoltammetries (right panel) of different Cu@Sn concentrations. $Cu_{90}Sn_{10}$ shows the lowest overpotential.

* These authors contributed equally to the work
† paolo.restuccia@unibo.it
‡ raffaello.mazzaro@unibo.it

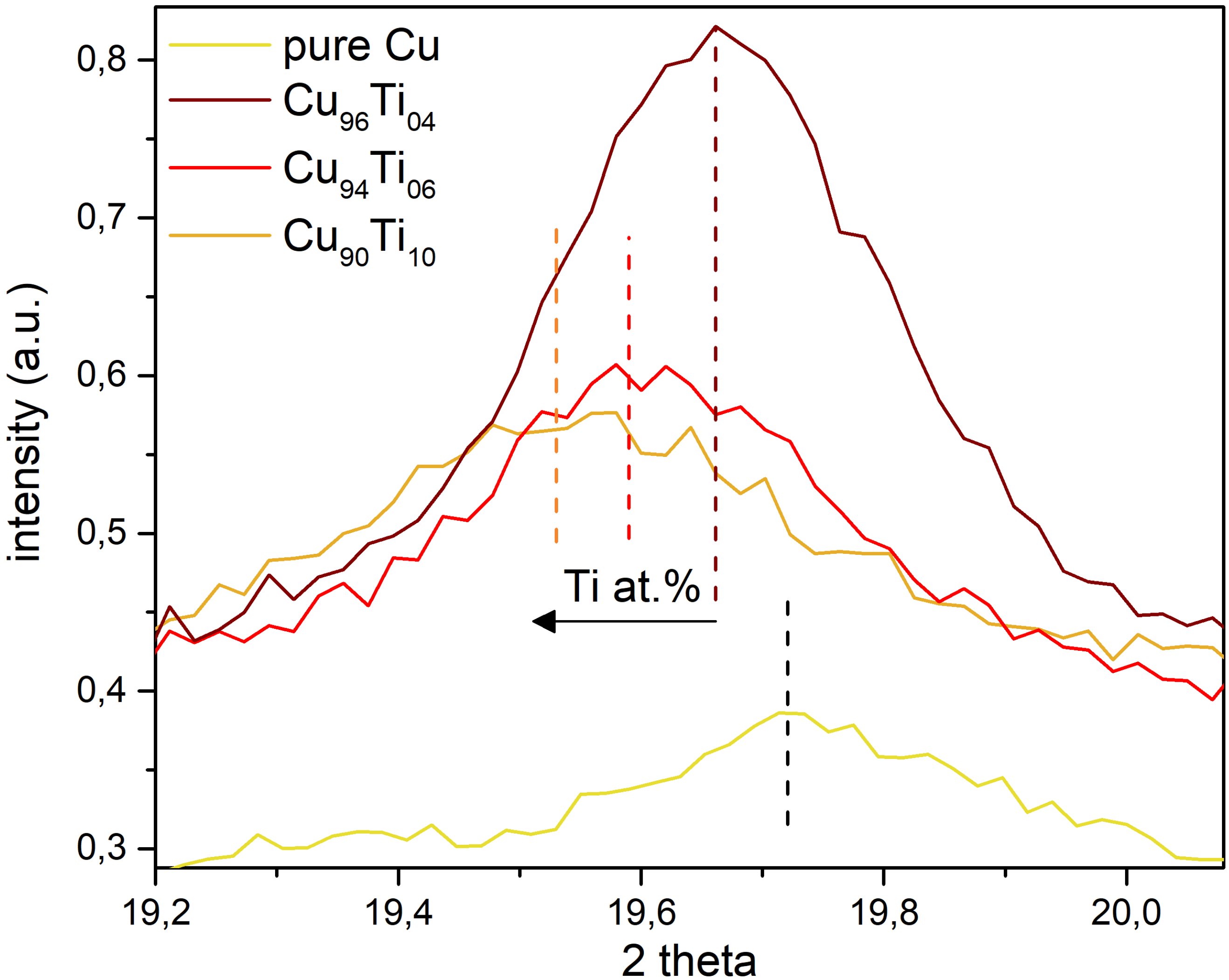

FIG. S3: XRD data (Mo kα) of Cu and Cu@Ti electrodes before EC tests. Increasing the Ti at.% the peak shifts towards lower angles.

TABLE S1: Applied potentials (V vs. RHE) recorded during chronopotentiometric measurements for all materials at current density values of 1, 5, and 10 mA.

	Pt	Ag	Cu	Cu@Ti	Cu@Sn	Ti	Sn
1 mA	−0.14	−0.31	−0.35	−0.32	−0.48	−0.64	−0.57
5 mA	−0.29	−0.53	−0.75	−0.45	−0.72	−1.07	−0.93
10 mA	−0.35	−0.63	−0.87	−0.58	−0.90	−1.32	−1.16

TABLE S2: Literature comparison of the potentials (V vs. RHE) measured at -10 mA/cm^2 for the Cu, Cu@Ti, and Cu@Sn electrodes.

	This work	Literature
Cu	−0.62	−0.63 [1]
Cu@Ti	−0.36	−0.35 [2]
Cu@Sn	−0.74	−0.77 [1]

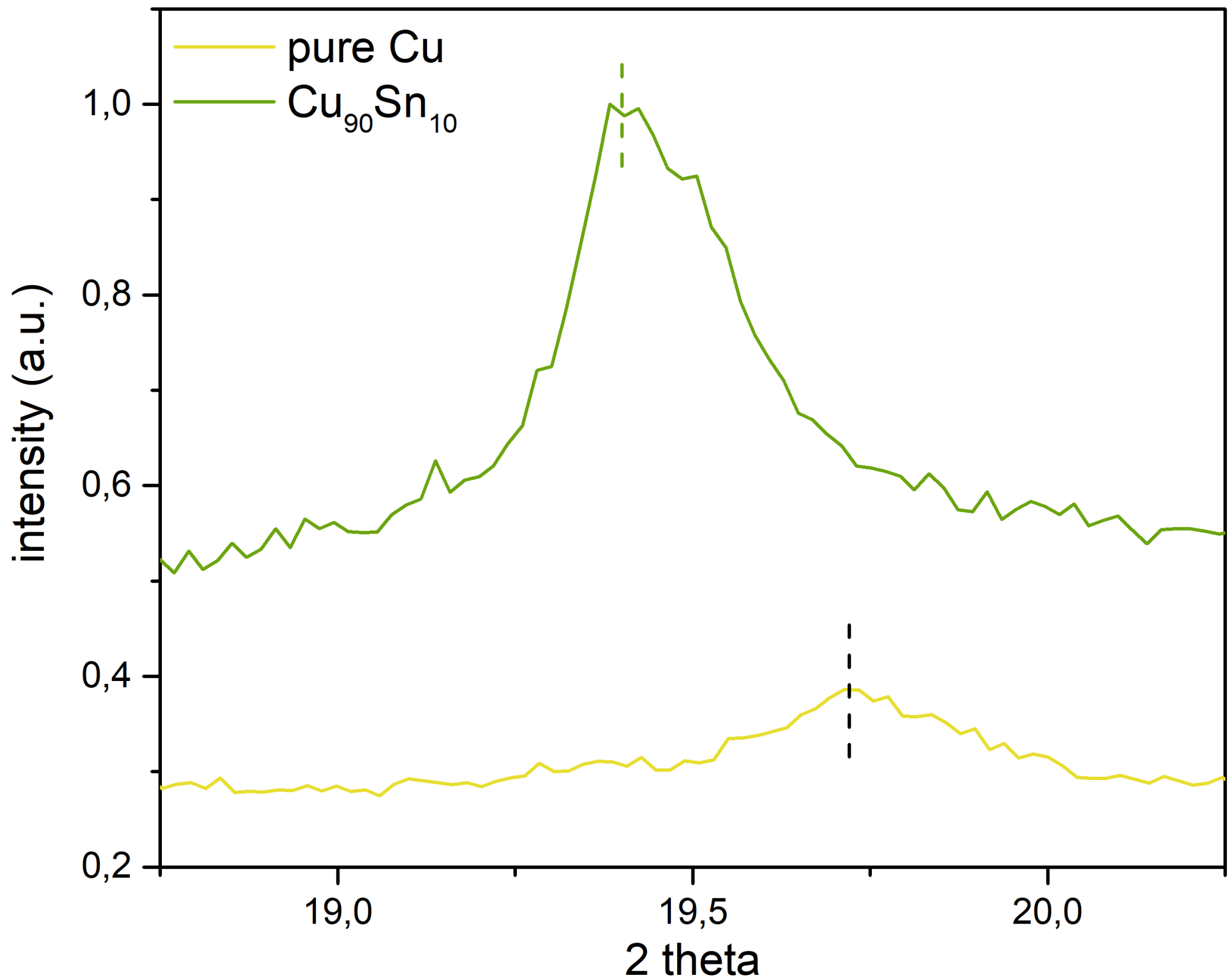

FIG. S4: XRD data (Mo kα) of Cu and Cu@Sn electrodes before EC tests. The presence of tin in the sample expands the copper lattice.

TABLE S3: Peak area values obtained from the μ-GC at 1 mA. Corresponding mole percentages relative to CO_2 content are included, based on prior calibration.

	Pt	Ag	Cu	Cu@Ti	Cu@Sn	Ti	Sn
H_2 peak area	6130	3500	4000	5600	3000	5721	1400
CO peak area	–	150000	2275	–	35500	5000	2000
CH_4 peak area	–	–	600	–	–	–	–
% H_2 produced	4.86	2.82	3.22	4.46	2.43	4.55	1.14
% CO produced	–	1.72	0.02	–	0.41	0.05	0.02
% CH_4 produced	–	–	0.003	–	–	–	–

TABLE S4: Peak area values obtained from the μ-GC at 5 mA. Corresponding mole percentages relative to CO_2 content are included, based on prior calibration.

	Pt	Ag	Cu	Cu@Ti	Cu@Sn	Ti	Sn
H_2 peak area	36800	21000	28000	30000	20000	26485	4677
CO peak area	–	410000	160000	–	150000	2900	1800
CH_4 peak area	–	–	4400	–	–	–	–
% H_2 produced	23.40	15.06	19.06	20.12	14.44	18.24	3.74
% CO produced	–	4.58	1.84	–	1.72	0.03	0.02
% CH_4 produced	–	–	0.028	–	–	–	–

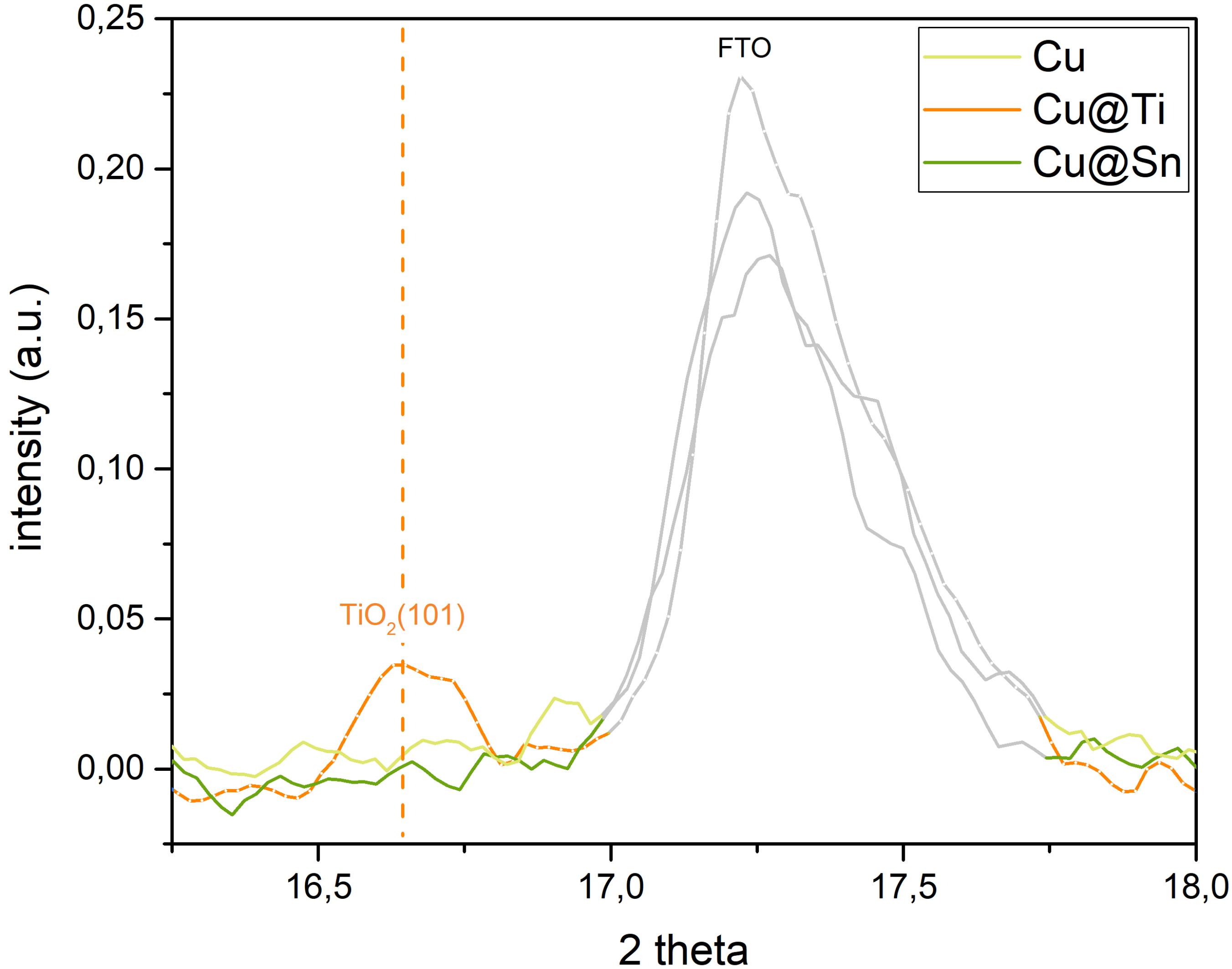

FIG. S5: XRD data (Mo kα) of the electrodes. Rutile ($TiO_2(101)$) peak present (orange dotted line) in the Cu@Ti electrode and not present in the other samples

TABLE S5: Peak area values obtained from the μ-GC at 10 mA. Corresponding mole percentages relative to CO_2 content are included, based on prior calibration.

	Pt	Ag	Cu	Cu@Ti	Cu@Sn	Ti	Sn
H_2 peak area	70000	55000	58000	69000	53000	60655	25000
CO peak area	–	375000	75000	–	200000	6250	24800
CH_4 peak area	–	–	2250	–	–	–	–
% H_2 produced	32.54	29.81	30.54	32.40	29.27	31.11	17.40
% CO produced	–	4.20	0.87	–	2.28	0.07	0.28
% CH_4 produced	–	–	0.015	–	–	–	–

II. DFT ADSORPTION ENERGIES

Fig. S6 shows the adsorption energy gains for all the considered intermediates on the different substrates. Ti(0001) and Cu(111) provided respectively the highest and lowest adsorption energies for all the intermediates, apart from CH_4. These results are consistent with the calculated surface energies and show trends that are comparable with similar systems found in literature, as in the case of Cu(111) [3, 4]. Although complete data on the adsorption of

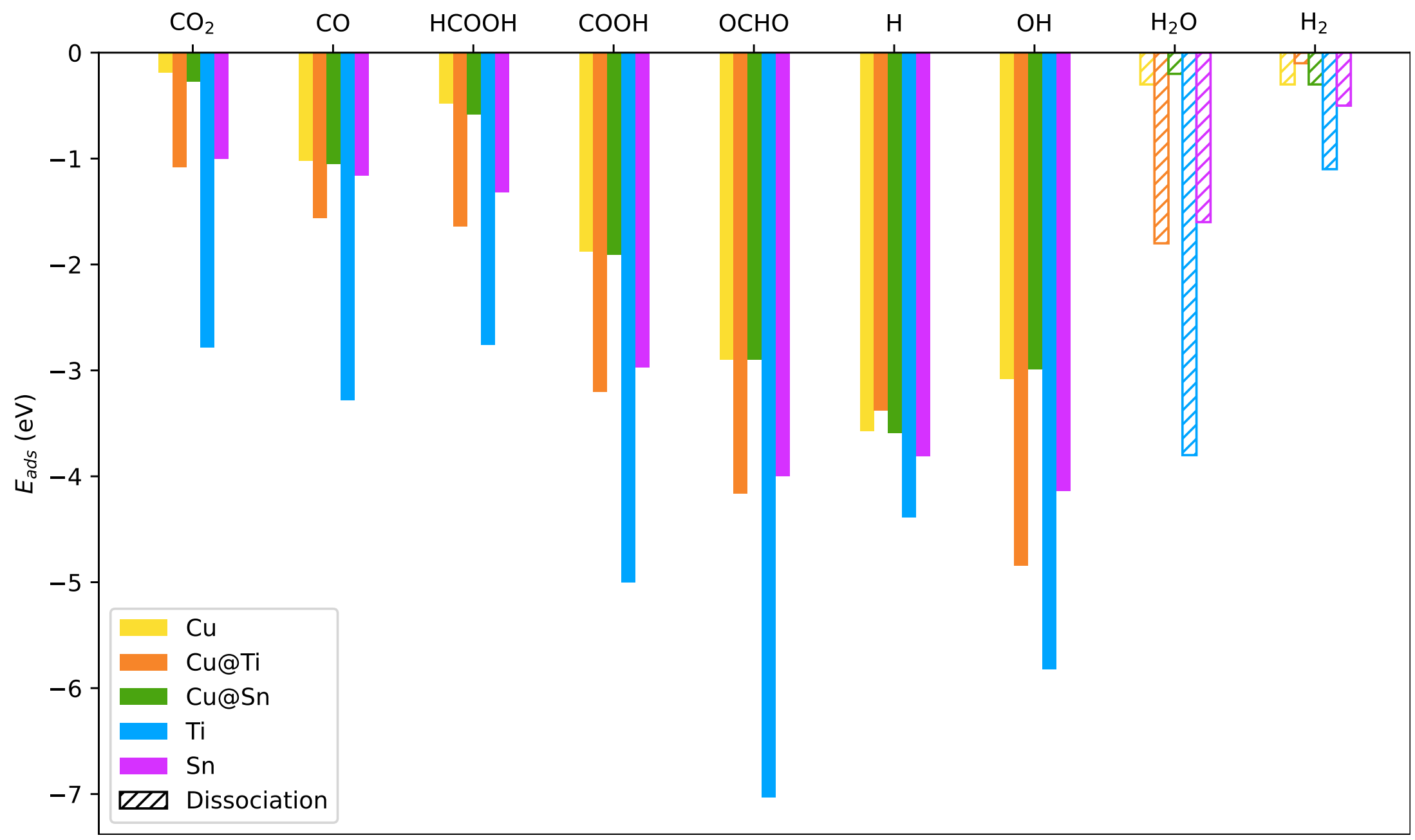

FIG. S6: Adsorption (solid colours) and dissociation (dashed areas) energies of the molecular intermediates of interest on the different surfaces. Yellow represents the energetics on Cu(111), red is for Ti(0001), orange for Ti@Cu(111), blue for Sn(111), and green represents Sn@Cu(111). Greater negative values correspond to more favourable adsorption/dissociation energy.

several CO_2RR key intermediates are unavailable for the remaining surfaces, our data align well with previous works. For instance, the adsorption energies of CO_2, CO, and CH_4 agree with data for titanium and anatase [5, 6]. Similarly, the CO_2 and CO adsorption energies for Ti@Cu(111) closely match those of an analogous SAA structure [7]. For Sn(111) and Sn@Cu(111), the adsorption energy trends align with observations on the Sn(211) surface and a similar bimetallic system [8].

Comparing the adsorption energies of CO_2RR intermediates across the different substrates, we can immediately notice that Sn(111) had a similar reactivity to Cu(111) despite its lower surface energy, as previously reported for the Sn(211) surface [8]. On the other hand, the adsorption energy obtained for the SAAs fell between those of their corresponding pristine surfaces, with two notable exceptions, as shown in Fig. S6. The first one is the adsorption of CH_4 on Ti@Cu(111) and Sn@Cu(111), which was more favourable than on the remaining surfaces. This effect is particularly evident for the Ti@Cu, where the synergy between copper and titanium can mitigate the ”methanephobia” process of pure Ti [6]. To investigate whether charge transfer contribute to this behaviour, we performed a Bader charge analysis employing the code developed by the group of Henkelman [9–12]. We found that the substituted Ti and Sn in the SAAs exhibited charge depletions of approximately $+1.25e$ and $+0.21e$, respectively, when interacting with all molecular fragments. These values were significantly higher than those of the corresponding surface atoms in the pristine substrates ($+0.23e$ for Ti and $+0.09e$ for Sn). In both Ti@Cu and Sn@Cu, methane exhibited a mild charge accumulation at the molecule-substrate interface ($-0.31e$ on C for Ti@Cu, $-0.06e$ on the H facing the Sn@Cu substrate), whereas on the bare substrates, CH_4 remained nearly neutral or showed a slight charge depletion. This increased electrostatic interaction explains the stronger CH_4 adsorption on Ti@Cu and Sn@Cu. The second exception is the adsorption of H on Ti@Cu(111), which is less favourable than on either of the pristine surfaces. In the pristine alloy, the Ti dopant donates about $+1.25e$ to neighboring Cu atoms. Upon H adsorption, this charge distribution is perturbed, and the nearby Cu atoms lose part of their acquired negative charge. The Ti atom remains positively charged, but the resulting local electrostatic environment weakens its ability to stabilize the negatively charged H adatom, explaining its weaker binding compared to pure Cu or Ti surfaces. Notably, when H adsorbed on the Ti@Cu substrate, the Ti–H bond length (1.94 Å) was longer than the Cu–H bond (1.75 Å), suggesting a weaker interaction over the Ti atoms.

The previous adsorption energies indicate which are the most attractive substrates for the different molecular species. However, they could not unravel possible dissociative mechanisms occurring at the interface between the catalyst and the electrolyte. For example, a highly negative Gibbs free energy in the HER step implies a highly favourable H_2 dissociative adsorption that can potentially poison the substrate, thus hindering the CO_2RR. On the

other hand, many catalytic experiments are performed in aqueous environments, where Ti and Sn substrate can easily undergo oxidation. Therefore, we evaluated the dissociation energies of H_2O and H_2 on the different surfaces and compare the associated energy gains to those calculated for the molecular adsorption of CO and other species in Fig. S6.

The results show that the dissociation of both H_2 and H_2O are more favourable on pristine titanium and tin than on the other substrates, which is consistent with the high oxidative tendency of both Ti and Sn [13, 14]. This effect could be significantly mitigated by employing SAAs, which increased the H_2O dissociation energy on Ti@Cu by a factor of 2 and by almost one order of magnitude for the Sn@Cu substrate. Similar variations were obtained for the H_2 dissociation energy, which had a 2-fold increase over Sn@Cu and a one order of magnitude increment on the Ti@Cu substrate. By comparing these data with the CO adsorption energy, as an indicator of the substrate tendency towards CO_2RR, we identified Cu(111) and Sn@Cu(111) as the most promising candidates for the reaction.

III. POTENTIAL-DEPENDENT HER FREE ENERGY DIAGRAM

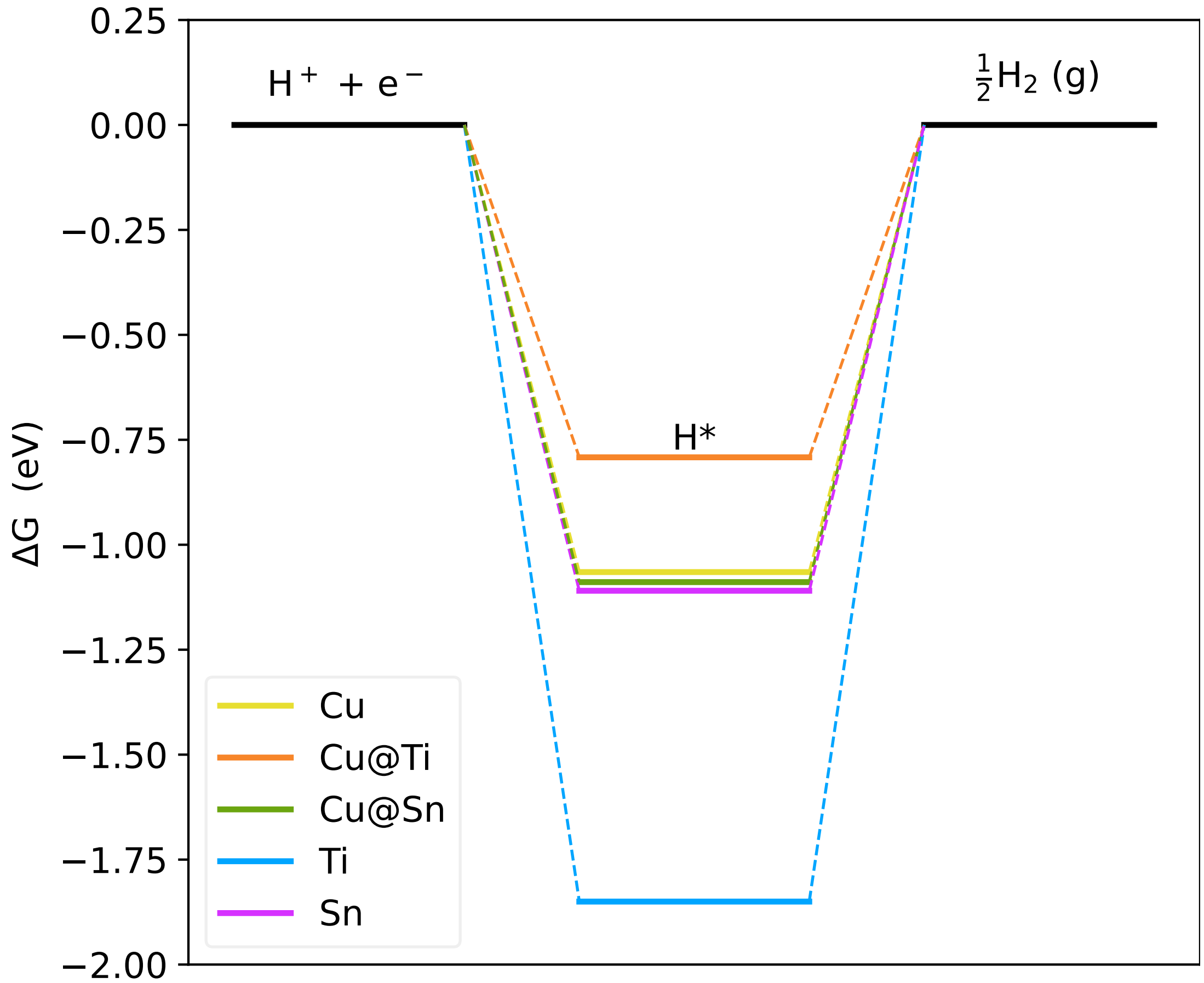

FIG. S7: Free energy diagram of HER at -0.9 V vs SHE for the different surfaces

IV. CALCULATED TOTAL ENERGY VALUES AND FREE ENERGY CONTRIBUTIONS

Molecule	E (eV)	ZPE (eV)	TS (eV)	G (eV)
H_2	-6.76	0.27	0.41	-6.81
CO	-14.79	0.13	0.62	-15.19
CO_2	-22.99	0.31	0.67	-23.24
HCOOH	-29.92	0.89	0.77	-29.69
H_2O	-14.22	0.57	0.67	-14.22

TABLE S6: Total energy E, ZPE, entropic contribution TS, and total free energy G of the isolated molecules at $T = 300$ K and $P = 1$ atm ($P_{H_2O} = 0.035$ atm).

Surface	E_{surf} (eV)
Cu(111)	-78.51
Ti(0001)	-255.23
Sn(111)	-89.82
Cu@Ti	-180.52
Cu@Sn	-177.38

TABLE S7: Total energies (E_{surf}) of the clean slabs.

Cu(111) adsorbate	E (eV)	ZPE (eV)	TS (eV)	G (eV)
CO*	-94.47	0.17	0.16	-94.37
CO_2*	-101.82	0.31	0.20	-101.63
COOH*	-105.01	0.60	0.24	-104.53
HCOOH* (CH_2O_2*)	-109.10	0.89	0.31	-108.38
H*	-82.25	0.17	0.01	-82.08
OCHO*	-105.75	0.61	0.17	-105.22
TS(COOH*)	-103.88	0.38	0.25	-103.64
TS(OCHO*)	-104.33	0.43	0.21	-104.33

TABLE S8: E, ZPE, TS, and G for adsorbates on Cu(111) at $T = 300$ K.

Ti(0001) adsorbate	E (eV)	ZPE (eV)	TS (eV)	G (eV)
CO*	-273.92	0.19	0.07	-273.75
CO_2*	-281.61	0.27	0.11	-281.39
COOH*	-285.24	0.56	0.17	-284.75
HCOOH* (CH_2O_2*)	-288.48	0.84	0.20	-287.74
H*	-259.76	0.18	0.00	-259.58
OCHO*	-286.99	0.59	0.12	-286.44
TS(COOH*)	-284.11	0.37	0.15	-283.80

TABLE S9: E, ZPE, TS, and G for adsorbates on Ti(0001) at $T = 300$ K.

Sn(111) adsorbate	E (eV)	ZPE (eV)	TS (eV)	G (eV)
CO*	-105.69	0.16	0.08	-105.56
CO_2*	-113.65	0.31	0.20	-113.46
COOH*	-117.01	0.60	0.21	-116.52
HCOOH* (CH_2O_2*)	-120.95	0.89	0.27	-120.21
H*	-93.59	0.16	0.02	-93.43
OCHO*	-117.77	0.60	0.27	-117.32
TS(COOH*)	-115.26	0.38	0.18	-114.96
TS(OCHO*)	-116.19	0.47	0.16	-115.80

TABLE S10: E, ZPE, TS, and G for adsorbates on Sn(111) at $T = 300$ K.

Cu@Ti adsorbate	E (eV)	ZPE (eV)	TS (eV)	G (eV)
CO*	-197.08	0.18	0.16	-196.98
CO_2*	-204.83	0.29	0.20	-204.64
COOH*	-208.30	0.61	0.22	-207.80
HCOOH* (CH_2O_2*)	-212.25	0.89	0.26	-211.50
H*	-183.99	0.17	0.01	-183.82
OCHO*	-208.97	0.61	0.23	-208.48
TS(COOH*)	-207.67	0.39	0.13	-207.33
TS(OCHO*)	-207.96	0.45	0.15	-207.59

TABLE S11: E, ZPE, TS, and G for adsorbates on Cu@Ti at $T = 300$ K.

Cu@Sn adsorbate	E (eV)	ZPE (eV)	TS (eV)	G (eV)
CO*	-193.40	0.18	0.15	-193.29
CO_2*	-200.72	0.31	0.20	-200.53
COOH*	-203.86	0.59	0.24	-203.39
HCOOH* (CH_2O_2*)	-208.00	0.89	0.31	-207.29
H*	-181.15	0.18	0.00	-180.97
OCHO*	-204.54	0.61	0.23	-204.05

TABLE S12: E, ZPE, TS, and G for adsorbates on Cu@Sn at $T = 300$ K.

Step (ΔG from reference, eV)	Cu(111)	Ti(0001)	Sn(111)	Cu@Ti	Cu@Sn
**CO pathway**					
1: CO_2 + * + 2(H^++e^-)	0.00	0.00	0.00	0.00	0.00
2: CO_2* + 2(H^++e^-)	0.13	-2.91	-0.39	-0.87	0.10
TS: $CO_2^* \rightarrow COOH*$	1.53	-1.93	1.51	-0.16	1.53
3: COOH* + (H^++e^-)	0.63	-2.87	-0.05	-0.63	0.64
4: CO* + H_2O	-0.03	-2.69	0.09	-0.63	-0.08
5: CO + * + H_2O	0.64	0.64	0.64	0.64	0.64
**HCOOH pathway**					
1: CO_2 + * + 2(H^++e^-)	0.00	0.00	0.00	0.00	0.00
2: CO_2* + 2(H^++e^-)	0.13	-2.91	-0.39	-0.87	0.10
TS: $CO_2^* \rightarrow OCHO*$	0.84	-	0.67	-0.41	0.84
3.1: OCHO* + (H^++e^-)	-0.06	-4.57	-0.85	-1.31	-0.01
4.1: HCOOH*	0.19	-2.45	-0.34	-0.92	0.14
5.1: HCOOH + *	0.36	0.36	0.36	0.36	0.36
**HER**					
1: $\frac{1}{2}H_2$	0.00	0.00	0.00	0.00	0.00
2: H*	-0.16	-0.95	-0.21	0.11	-0.19
**HER - Tafel step**					
1: H* + H*	0.00	0.00	0.00	0.00	0.00
2: TS	0.78	-	0.80	0.50	0.78
3: H_2(g)	0.22	1.87	0.08	0.35	0.22

TABLE S13: ΔG values (eV) referenced to the chosen reference state for each pathway.

[1] W. Ren, X. Tan, J. Qu, S. Li, J. Li, X. Liu, S. Ringer, J. Cairney, K. Wang, S. Smith, and C. Zhao, Isolated copper–tin atomic interfaces tuning electrocatalytic co2 conversion, Nature Communications **12** (2021).

[2] Q. Lu, G. S. Hutchings, W. Yu, Y. Zhou, R. V. Forest, R. Tao, J. Rosen, B. T. Yonemoto, Z. Cao, H. Zheng, J. Q. Xiao, F. Jiao, and J. G. Chen, Highly porous non-precious bimetallic electrocatalysts for efficient hydrogen evolution, Nature Communications **6**, 6567 (2015).

[3] E. O. Bartaquim, R. C. Bezerra, A. F. B. Bittencourt, and J. L. F. Da Silva, Computational investigation of van der waals corrections in the adsorption properties of molecules on the Cu(111) surface, Phys. Chem. Chem. Phys. **24**, 20294 (2022).

[4] Y. Guan, W. Suo, Z. Zhang, Y. Wang, S. Sun, and G. Liu, Insights on the catalytic active site for CO_2 reduction on copper-based catalyst: A DFT study, Molecular Catalysis **511**, 111725 (2021).

[5] S. Huygh, A. Bogaerts, and E. C. Neyts, How oxygen vacancies activate CO_2 dissociation on TiO_2 anatase (001), The Journal of Physical Chemistry C **120**, 21659 (2016).

[6] J. R. de Arellano, I. Carrillo, and L. Magaña, Chemisorption of carbon monoxide, carbon dioxide and "methanephobia" on the [0001] titanium surface, Applied Surface Science **478**, 128 (2019).

[7] J.-C. Jiang, J.-C. Chen, M.-d. Zhao, Q. Yu, Y.-G. Wang, and J. Li, Rational design of copper-based single-atom alloy catalysts for electrochemical CO_2 reduction, Nano Research **15**, 7116 (2022).

[8] S. Li, S. Sun, W. Suo, G. Liu, G. Wang, Y. Wang, J. Li, and Z. Zhang, Theoretical investigation of electrochemical reduction mechanism of CO_2 on the Cu(111), Sn@Cu(111) and Sn(211) surfaces, Applied Surface Science **564**, 150418 (2021).

[9] W. Tang, E. Sanville, and G. Henkelman, A grid-based bader analysis algorithm without lattice bias, Journal of Physics: Condensed Matter **21**, 084204 (2009).

[10] E. Sanville, S. D. Kenny, R. Smith, and G. Henkelman, Improved grid-based algorithm for bader charge allocation, Journal of Computational Chemistry **28**, 899 (2007).

[11] G. Henkelman, A. Arnaldsson, and H. Jónsson, A fast and robust algorithm for bader decomposition of charge density, Computational Materials Science **36**, 354 (2006).

[12] M. Yu and D. R. Trinkle, Accurate and efficient algorithm for bader charge integration, The Journal of Chemical Physics **134**, 064111 (2011).

[13] J. Liu, X. Fan, C. Sun, and W. Zhu, Oxidation of the titanium(0001) surface: diffusion processes of oxygen from DFT, RSC Adv. **6**, 71311 (2016).

[14] J. Martinez Medina, A. Philippe, J. Guillot, C. Vergne, Y. Fleming, and E. Barborini, Intermediate tin oxide in stable core-shell structures by room temperature oxidation of cluster-assembled nanostructured Sn films, Applied Surface Science **658**, 159846 (2024).
